# Collagen-stimulating activity of a pentapeptide conjugate and self-assembly into micelles

**DOI:** 10.1039/d6nr03203f

**Published:** 2026-10-26

**Authors:** Lucas de Mello, Valeria Castelletto, Jani Seitsonen, Ian W. Hamley

**Affiliations:** a School of Chemistry, Food Biosciences and Pharmacy, University of Reading Whiteknights Reading RG6 6AD UK I.W.Hamley@reading.ac.uk; b Nanomicroscopy Center, Aalto University Puumiehenkuja 2 FIN-02150 Espoo Finland

## Abstract

We present a lipopeptide conjugate, farnesyl-mercapto(diethylene glycol)-KTTKS (FMdEG-KTTKS), designed to include the KTTKS collagen-stimulating sequence attached to a terpenoid branched lipid chain *via* a short hydrophilic spacer. This conjugate stimulates fibroblasts to produce greater than 200% more collagen per cell than conventional stimulants. This activity is observed at low concentration, well below a critical micelle concentration (CMC) determined by fluorescence probe measurements. Above the CMC, this molecule self-assembles into well-defined spherical micelles, as shown by cryogenic-transmission electron microscopy (cryo-TEM) and small-angle X-ray scattering (SAXS). The behaviour of this conjugate is contrasted with that of related lipopeptides: FMdEG-KTTKS forms micelles across a wide pH range, instead of nanotapes observed for C_16_-KTTKS (Matrixyl™). The molecule is shown to stimulate production of type I collagen (beneficial for anti-aging treatments) and promotes repopulation of fibroblasts in a cell monolayer after scratching, as a simple model for skin injury. FMdEG-KTTKS also has anti-inflammatory activity, reducing the production of nitrite in macrophages challenged with bacterial lipopolysaccharide.

## Abstract 2

A lipopeptide conjugate, farnesyl-mercapto(diethylene glycol)-KTTKS (FMdEG-KTTKS), with a KTTKS collagen-stimulating sequence attached to a terpenoid branched lipid chain, forms micelles and stimulates collagen production in cells.

## Graphical abstract

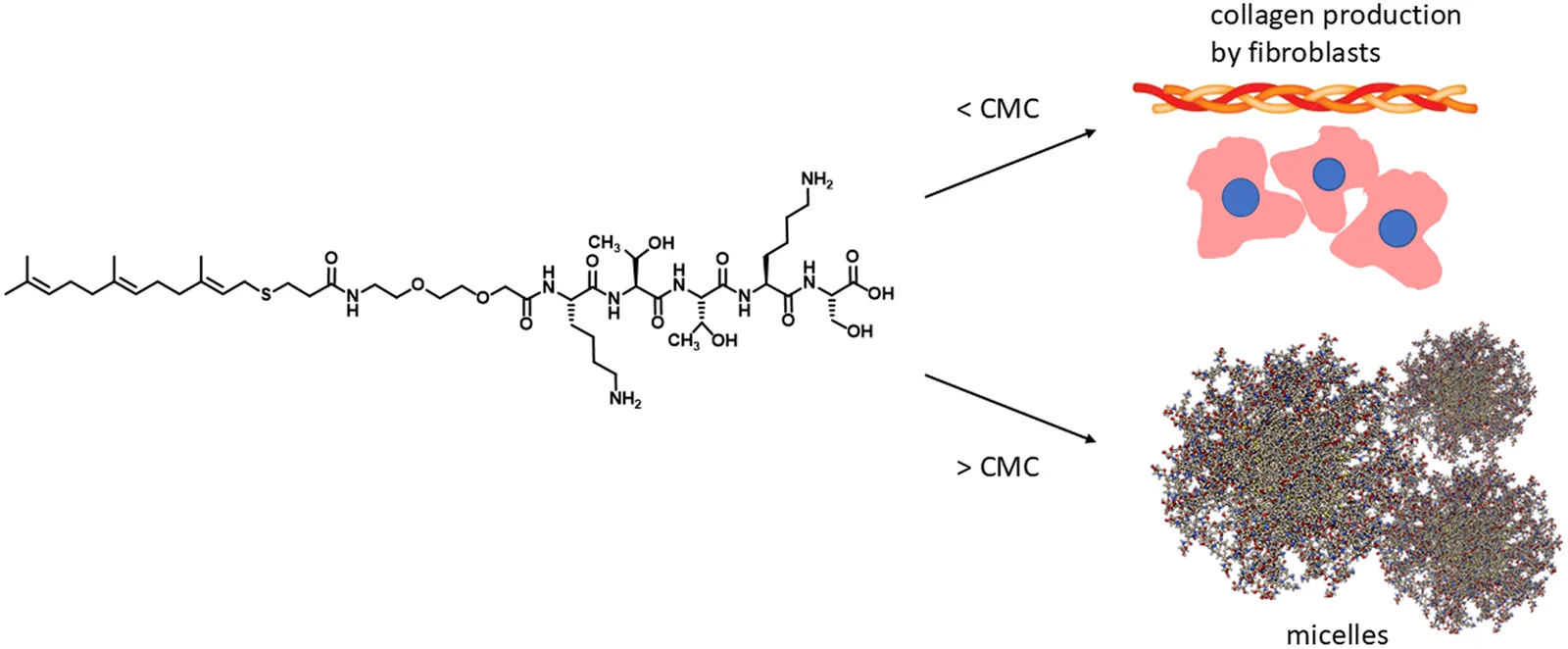

## Introduction

There is great interest in peptide-based bioactive molecules with applications including tissue engineering and regenerative medicine. Peptides and their conjugates have been developed for wound healing and cosmetics based on the stimulation of extracellular matrix (ECM) production. A particularly important component of the ECM is collagen, and peptides that stimulate the synthesis of collagen have attracted a great deal of interest in the cosmetics industry. Numerous studies have demonstrated that cells such as fibroblasts can respond to bioactive peptides, especially those derived from ECM fragments, such as collagen or elastin-derived peptides, which act as cell signalling molecules called ‘matrikines’.^1^ Matrikines are fragments of ECM, usually short peptide sequences, liberated by partial proteolysis of the ECM and modulate many processes related to the repair or homeostasis of affected tissues, such as immune function, ECM restoration, angiogenesis, and cell growth.^2^

One well established example of a peptide in the market commercialized as an anti-aging active-compound is the KTTKS pentapeptide, which has been incorporated into the lipopeptide (peptide amphiphile, PA) C_16_-KTTKS within the Matrixyl™ family, developed by Sederma. The KTTKS pentapeptide is based on a sequence taken from a propeptide present in the Human type I collagen sequence.^3,4^ It has been reported that both the peptide KTTKS^3^ and C_16_-KTTKS^5^ are able to promote type I collagen biosynthesis *in vitro*. Studies also suggest that C_16_-KTTKS can reduce the appearance of facial wrinkles and may help in the recovery of skin wounds.^6–8^ Although it has been established that KTTKS improves the production of type I collagen *in vitro*, an important consideration in the topical application of peptide amphiphiles is how to improve the transport of the bioactive peptide across the stratum corneum, a natural barrier against exogenous compounds, and the lipidated conjugate was developed to improve delivery across the epidermis. In prior work, our group demonstrated that C_16_-KTTKS self-assembles into highly extended nanotapes, based on a bilayer packing of the lipopeptide molecules.^9^ We also investigated the self-assembly of the related PAs C_14_-KTTKS and C_18_-KTTKS with distinct lipid chain lengths,^10^ as well as that of a lipopeptide with shorter peptide sequences C_16_-KT and compared the self-assembly of this new lipopeptide with other molecules relevant to skincare applications.^11^ Our group also examined the pH-dependent self-assembly of C_16_-KTTKS and it was found to form micelles at low pH in contrast to extended nanotapes at higher pH.^12^ Recently, we have compared the pH-dependent self-assembly and bioactivity of C_16_-KTTKS and the arginine-substituted derivative C_16_-RTTRS, including a comparison for two salt forms.^13^ The Hamley group also introduced the homologue bearing anionic glutamic acid residues instead of cationic lysine residues, C_16_-ETTES,^14^ and investigated its self-assembly into nanotapes as well as its co-assembly with C_16_-KTTKS.^14^ It was also used as a diluent in mixtures with a lipopeptide bearing an RGD integrin-binding motif, and the structure and conformation of the co-assemblies was studied in detail.^15^ The mixtures were shown to represent promising systems for cell culture and tissue engineering, forming stable coatings with good adhesion, proliferation and prolonged viability of human corneal stromal fibroblasts (hCSFs), while improving the cell phenotype.^16^ They could also form stable coatings on aligned hydrophobic substrates, creating aligned extracellular matrix.^16^ More recently, we studied the self-assembly and bioactivity of a conjugate of KTTKS to lipoic acid. The N-terminal lipoyl group confers redox activity (chemical and photoinduced) through reduction of the disulfide bond.^17^ Lipoyl-KTTKS has excellent cytocompatibility across a wide concentration range, stimulates collagen production and enhances the rate of cell coverage in a simple *in vitro* scratch assay of “wound healing”.^17^ We also introduced lipopeptides bearing one or two cycloalkane chains (cycloheptadecyl or cyclododecyl) conjugated to KTTKS and studied self-assembly and bioactivity, including *in vivo* wound healing studies.^18^ The conjugate to two cyclododecyl lipids showed particularly promising collagen deposition and wound healing properties including promotion of vascularization. KTTKS derivatives show great potential to enhance the biocompatibility and delivery of KTTKS and its analogues to living cells and to target cellular processes.

Attachment of branched chains to proteins/peptides *via* prenylation is a post-translational modification which has evolved to enhance cell membrane binding. One type of terpenoid (isoprenoid) group added in this way is a farnesyl chain, in the process of farnesylation. It is of interest to attach such chains synthetically to bioactive peptides to promote binding to cell membranes, but also due to the use of farnesol-based compounds in cosmetic products. Farnesol is an essential oil derived from many plants and flowers that is used in cosmetics and fragrances^19^ and has been proposed as a skin permeation enhancer.^20–22^ This inspired Mortazavi *et al.* to conjugate the terpenoids citronellic acid or perillic acid to KTTKS to enhance permeation across the epidermis.^23^

Here, a novel conjugate was designed with by combining a branched farnesyl lipid chain along with a mercapto-di(ethylene glycol) linker, intended to improve the solubility in the conjugate called FMdEG-KTTKS. The distinct N-terminal moiety compared to simple unbranched lipids leads to self-assembly into spherical micelles, contrasting with β-sheet nanotape formation of the C_16_-KTTKS or C_18_-KTTKS^10^ lipopeptides (C_14_-KTTKS showed presented an unordered conformation^10^). Notably, FMdEG-KTTKS stimulates more than double the production of collagen per fibroblast cell compared to conventional stimulants. Collagen-specific dye staining as well as immunocytochemistry using antibodies specific to collagen type I and III, provide detailed information on collagen production and distribution. We also show that FMdEG-KTTKS performs as an anti-inflammatory molecule, reducing nitrite production by macrophages after exposure to bacterial lipopolysaccharide. To further assess relevance to wound healing, scratch assays of cell monolayers were performed, which indicate that the lipopeptide acts to promote fibroblast repopulation as a simple model of wound healing. Together, these findings position FMdEG-KTTKS as a versatile candidate for potential future applications in cosmetics due to collagen-stimulating properties, and in regenerative medicine due as it promotes wound healing.

## Experimental

### Materials

Conjugate Farnesyl-mercaptopropyl-(EG)_2_-KTTKS (EG: ethylene glycol), denoted FMdEG-KTTKS, was synthesized using automated solid phase Fmoc peptide synthesis by Peptide Protein Research Ltd (UK). The product was purified in acetonitrile and water containing 0.1% trifluoroacetic acid (TFA) prior to lyophilisation. FMdEG-KTTKS is present as its TFA acid salt in solution. Several batches were purchased and purity for all was >95% by analytical high-performance liquid chromatography (HPLC) (data for one batch in Fig. S1). Measurements were performed using an Agilent 1100 HPLC with Kinetex XB-C18 2.6 μm 100 Å column at a flow rate 1.5 ml min^−1^ (injection volume: 20 μl), with gradient from 10%–90% acetonitrile, at 60 °C, with a sample dissolved in DMSO. Electro-spray ionization (ESI-MS) mass spectrometry using an Agilent 1946D MS instrument was used to determine *M*_w_ 1001.6 Da (measured) (Fig. S2), 1001.3 Da (expected). Scheme 1 displays the chemical structure of FMdEG-KTTKS.

**Scheme 1:**
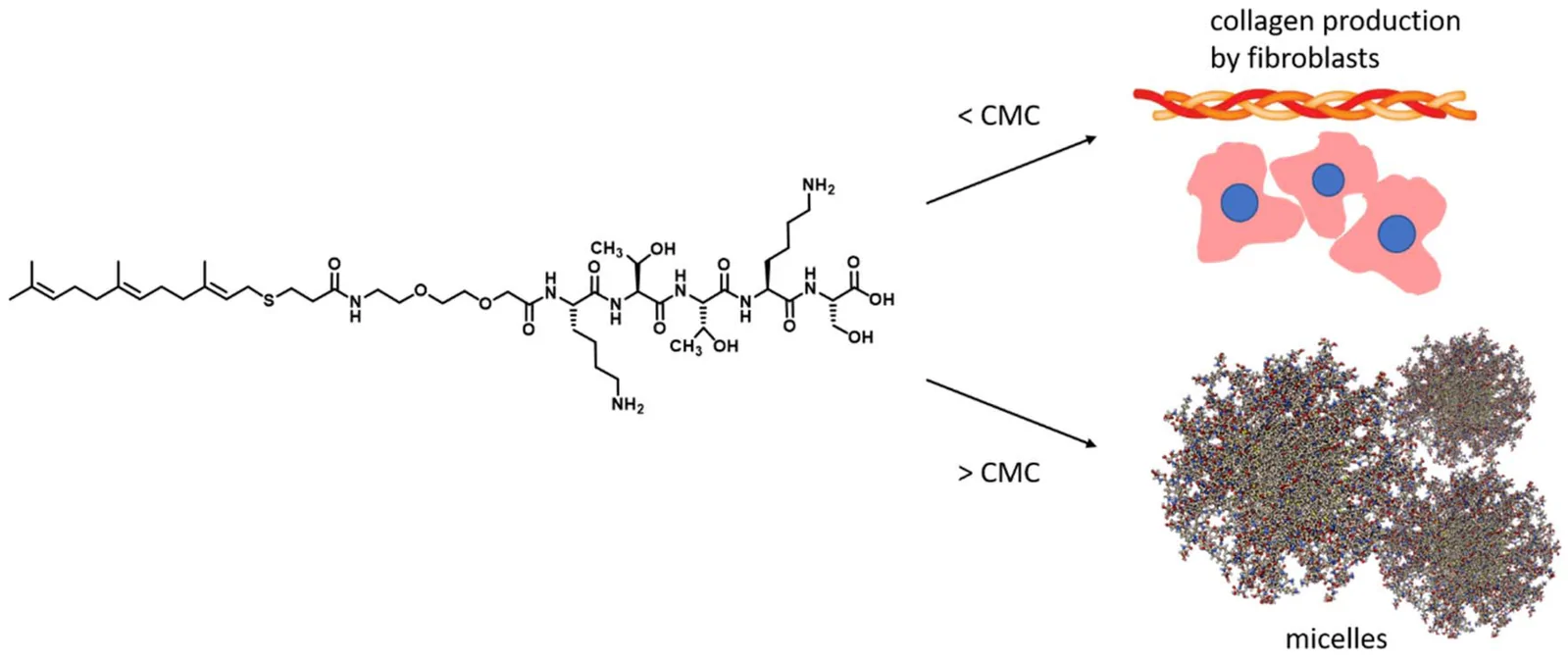
Chemical structure of FMDEG-KTTKS showing self-assembly above critical aggregation concentration and collagen-stimulating activity below.

All self-assembly and conformational studies were performed in water, with measured pH = 2.7 for 1 wt% sample (pH = 3.4 for 0.1 wt% sample). For cell studies, the pH was maintained around 7.2–7.4, as indicated by the phenol red present in media.

### Fluorescence spectroscopy

Experiments were carried out using a 10.0 mm × 5.00 mm quartz cell in a Varian Model Cary Eclipse spectrofluorometer (Agilent, UK). The critical micelle concentration (CMC) was determined by titration of the fluorescent dye 8-anilino-1-naphthalene sulfonic acid (ANS). A mother solution of 0.2 wt% FMdEG-KTTKS was prepared using 2 × 10^−3^ wt% ANS as a solvent. Dilutions in the rage 0.15–2 × 10^−3^ wt% FMdEG-KTTKS were prepared from the mother solution, by adding weighed amounts of 2 × 10^−3^ wt% ANS. The fluorescence emission spectra of the sequence of dilutions were recorded between 400 and 670 nm exciting the sample at 356 nm.

### Dynamic light scattering

DLS measurements were carried out using an ALV/CGS-3 Compact Goniometer System with ALV/LSE-5003 correlator (ALV, Langen, Germany) using vertical polarized incident light of wavelength *λ* = 632.8 nm. Measurements were performed at *θ* = 90° to the incident beam, and data were collected three times for 30 s. The intensity autocorrelation functions were analyzed by a regularization method. The number-averaged size distribution was calculated from the autocorrelation function. The hydrodynamic radius, *R*_H_, was determined from the maxima in the size distribution *via* the Stokes–Einstein equation 
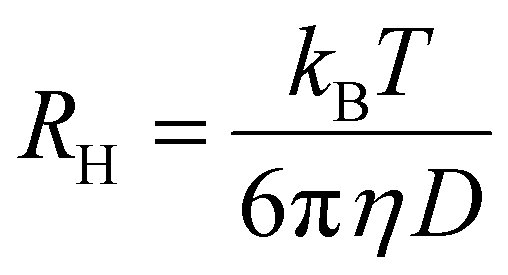
. Here *k*_B_ is the Boltzmann constant, *T* is temperature, *η* is solvent viscosity and *D* is the diffusion coefficient.

### Circular dichroism (CD) spectroscopy

CD spectra were recorded using a Chirascan spectropolarimeter (Applied Photophysics, Leatherhead, UK). Solutions were placed between parallel plates (0.01 mm or 0.1 mm path length). Spectra were measured with a 0.5 nm step, and 1 s collection time per step. The CD signal from the water background was subtracted from the CD data of the sample solutions. CD spectra were smoothed using the Chirascan Software for data analysis. The residue of the calculation was chosen to oscillate around the average, to avoid artifacts in the smoothed curve.

### Fourier transform infrared (FTIR) spectroscopy

Spectra were recorded using a ThermoScientific Nicolet iS5 instrument (ThermoFisher, UK) equipped with a DTGS detector, with a Specac Pearl liquid cell (Orpington, UK) with the sample contained between fixed CaF_2_ plates. Spectra were scanned 116 times over the range 900–4000 cm^−1^.

### Cryogenic-TEM (Cryo-TEM)

Imaging was carried out using a field emission cryo-electron microscope (JEOL JEM-3200FSC, Tokyo, Japan), operating at 200 kV. Images were taken in bright field mode and using zero loss energy filtering (omega type) with a slit width of 20 eV. Micrographs were recorded using a Gatan Ultrascan 4000 CCD camera. The specimen temperature was maintained at −187 °C during the imaging. Vitrified specimens were prepared using an automated FEI Vitrobot device using Quantifoil 3.5/1 holey carbon copper grids with a hole size of 3.5 μm. Just prior to use, grids were plasma cleaned using a Gatan Solarus 9500 plasma cleaner and then transferred into the environmental chamber of a FEI Vitrobot at room temperature and 100% humidity. Thereafter 3 μl of sample solution was applied on the grid and it was blotted twice for 5 seconds and then vitrified in a 1/1 mixture of liquid ethane and propane at a temperature −180 °C. The grids with vitrified sample solution were maintained at liquid nitrogen temperature and then cryo-transferred to the microscope.

### Small-angle X-ray scattering (SAXS)

Synchrotron SAXS experiments on solutions were performed using a BioSAXS robot on beamline BM29 at the ESRF (Grenoble, France).^24^ A few microlitres of samples were injected *via* an automated sample exchanger at a slow and very reproducible rate into a quartz capillary (1.8 mm internal diameter), in the X-ray beam. The quartz capillary was enclosed in a vacuum chamber, in order to avoid parasitic scattering. After the sample was injected in the capillary and reached the X-ray beam, the flow was stopped during the SAXS data acquisition. The *q* range was set to 0.004–0.4 Å^−1^, with *λ* = 1.03 Å. The images were captured using a Pilatus 1M detector. Data processing (background subtraction, radial averaging) was performed using dedicated beamline software ISPYB.

### SAXS models

All fitting was done using the software SASfit.^25^ The SAXS intensity was fitted using the form factor for a spherical shell, together with the structure factor for a hard sphere.^26,27^ The parameters for the spherical shell model are the overall radius of the spherical shell, *R*_1_, the radius of the core, *R*_2_, the scattering contrast of the inner core, *μ*, and the scattering contrast of the shell, *τ*. The parameters for the hard sphere model are the hard sphere radius, *R*_HS_, and the volume fraction of hard spheres, *ϕ*. The background for all the SAXS curves was fitted with a constant term, *C*.

### Cell culture

Human Dermal Fibroblasts from adult donors (HDFa) cells were obtained from SigmaAldrich (UK, product code 106-05A). For the cytotoxicity and collagen production assays, cells were cultivated in DMEM/F12 (ThermoFisher, UK) supplemented with 5% FBS (fetal bovine serum obtained from ThermoFisher, UK), Glutamax, and the antibiotics penicillin 100 IU mL^−1^, streptomycin 100 μg mL^−1^ and 0.25 μg mL^−1^ of the antimycotic amphotericin B (commercially known as Anti-Anti from Gibco, UK) and incubated at 37 °C within an atmosphere of 5% CO_2_.

### MTT cytocompatibility

MTT (3-(4,5-dimethylthiazol-2-yl)-2,5-diphenyltetrazolium bromide – obtained from SigmaAldrich, UK) is a yellow, water-soluble reagent. When incubated with living cells, the MTT is converted into insoluble chromogenic formazan crystals by the mitochondria present in the cytoplasm. Thus, the presence and number of living cells can be estimated by colorimetric assays using the conversion of MTT into formazan crystals.

For the MTT assay, cells were harvested with 0.25% trypsin/EDTA (Gibco, U.K) and seeded at a density of 1 × 10^4^ cells per well into 96 well plates in DMEM/F12 with 5% FBS, Glutamax, Anti-Anti and 5 µg mL^−1^ of insulin (SigmaAldrich, UK). Cells were incubated overnight at 37 °C under an atmosphere of 5% CO_2_. The following day, the media was replaced by DMEM/F12 with no supplements and six different concentrations of FMdEG-KTTKS. We also evaluated the cytotoxicity of conventional stimulants for collagen production (cells incubated with 1 mM ascorbic acid, 0.025 ng mL^−1^ TGFβ1 and 0.01 mg mL^−1^ insulin) and the control group was incubated only in DMEM/F12. After 3 days of incubation, cells were washed 3 times with PBS, and incubated for 4 hours with 0.5 mg mL^−1^ of MTT diluted in DMEM/F12 without phenol red (Invitrogen, UK) at 37 °C. After this, DMSO was added for the solubilization of the formazan crystals and the plate was incubated again for 15 minutes at 37 °C. After this final incubation, the absorbance at 560 nm was read using an Infinite F50 plate reader (Tecan, Switzerland). The data was analysed using the software Prism 8, first removing the background by subtracting the OD of control wells containing only the media with MTT, and estimating the number of cells by comparing the absorbance at 560 nm with controls incubated only in DMEM/F12 in triplicate. The statistical test applied was ordinary one-way ANOVA with Bonferroni's correction for multiple comparisons.

### Collagen production

To estimate the production of collagen by HDFa cells, 96 well plates were seeded at a density of 1 × 10^4^ cells per well in complete DMEM plus 5 µg mL^−1^ insulin and incubated for three days in DMEM without supplements in the presence of different concentrations of peptide and conventional stimulants (1 mM ascorbic acid, 0.025 ng mL^−1^ TGFβ1 and 0.01 mg mL^−1^ insulin). After this incubation, plates were washed with PBS and fixed using ice cold ethanol 70% to fix deposited material present on the well surfaces. This plate was transferred to a −80 °C ultra-freezer for 10 minutes and washed with water to remove any residual ethanol. After fixation, the collagen was incubated overnight in a solution containing Direct red 1 mg mL^−1^ in picric acid (Picrosirius red) at 4 °C under gentle agitation. Finally, the wells were washed with distilled water until all excess dye was removed, the collagen and cells were then treated with 1 mM NaOH for 10 minutes at room temperature and absorbance was obtained at 490 nm. This protocol follows that used previously by our group.^5^

### Polarized light microscopy

For microscopy, 5 × 10^4^ HDFa cells were deposited on coverslips and incubated in DMEM/F12 with 10% FBS, 2 mM glutamax, Anti-anti and 5 µg mL^−1^ insulin overnight in 24 well plates. The next day, the cells were washed and incubated for 3 days with the lipopeptide. Conventional stimulants and controls were incubated with only DMEM/F12 without any supplements. The coverslips were washed 3 times with PBS, fixed with cold ethanol and stained with Picrosirius red. After the incubation with the dye, the coverslips were washed with distilled water to remove any unbound dye and mounted in glass slides. The slides were imaged with an Olympus BX41microscope (Olympus Life Sciences, UK) equipped with polarizers. The methods used for this assay were based on previous reports.^5,28^

### Fibroblast immunocytochemistry

A quantity 9 × 10^4^ HDFa cells was incubated in 13 mm coverslips in 24 well plates using DMEM/F12 media containing 5% FBS, and was then left inside a cell incubator at 37 °C under an atmosphere of 5% CO_2_ over 24 hours to attach to the coverslips. For the assay, cells were incubated with DMEM/F12 containing conventional supplements, lipopeptide or only media for 72 hours inside the cell incubator. After 72-hour incubation, cells were washed 3 times with PBS to remove cellular debris and fixed using paraformaldehyde 4% (ThermoFisher, UK) for 10 minutes at room temperature, washed 3 times with PBS again and permeabilized with 0.1% Triton-X 100 in PBS (Merck, UK) for 15 minutes. The coverslips were again washed with PBS and blocked with BSA 1% for 1 hour at room temperature. Cells were washed again 3 times with PBS, with the third wash lasting for 5 minutes, and incubated with primary antibodies COL1A1 (E3E1X) mouse mAb for type I collagen and COL3A1 (E3K9U) or rabbit mAb for collagen type III (both from Cell Signaling, EU) at 37 °C for 1 hour. The coverslips were again washed 3 times with PBS for 5 minutes and incubated with secondary antibodies Goat anti-Mouse IgG-Alexa Fluor™ 488 and Donkey anti-Rabbit IgG-Alexa Fluor™ 647 (both from ThermoFisher, UK) for one hour at 37 °C protected from the light. Finally, cells were washed again and incubated for 1 hour with phalloidin–Texas red (ThermoFisher, UK), washed 3 times with PBS and mounted on glass slides using mounting media consisting of 90% glycerol + 10% PBS at pH 8.5 supplemented with ascorbic acid to prevent quenching. The nuclei of cells were stained using DAPI (ThermoFisher, UK). The images for immunofluorescence were acquired in an Alfie AR1 Confocal and processed using the free software Fiji-ImageJ. Lipopeptide was examined at a concentration of 0.0062 wt%. Control samples included DMEM without serum and positive controls consisted of DMEM containing the conventional stimulants TGF-β1, insulin and ascorbic acid.

### Anti-inflammatory activity (nitrite production)

To evaluate the anti-inflammatory effect of FMdEG-KTTKS, 5 × 10^3^ RAW 264.7 mouse macrophage cells were seeded in 96 well plates and incubated inside a cell incubator at 37 °C and 5% of CO_2_ for 24 hours. The medium used was DMEM (ThermoFisher, UK) supplemented with 10% FBS (Gibco, UK), Gibco™ Antibiotic-Antimycotic and l-glutamine (Gibco, UK). After the initial 24 hours incubation, RAW cells were washed 3 times with PBS and incubated in fresh DMEM without FBS or phenol red + 5 µg of *E. coli* lipopolysaccharide (Sigma-Aldrich, UK). Samples were incubated in the presence of lipopeptide at 0.025, 0.0125 and 0.00625 wt%. Control samples were incubated in basal media containing LPS. Negative controls were cultured in DMEM without LPS. For the non-control samples, after LPS incubation, 90 µL of supernatant from the cells was recovered and transferred into a new 96 well plate. In the new plate, 10 µL of Griess reagent solution (SigmaAldrich, UK, Cas-G4410) were added to each aliquot of supernatant and the plate was incubated at room temperature for 10 minutes protected from the light. The resulting absorbance was measured at 550 nm using a Tecan Infinite F50 plate reader.

### Scratch assay model of skin healing

For the scratch assay, L929 murine fibroblasts were cultivated in DMEM containing 10% FBS, Gibco™ Antibiotic-Antimycotic and l-glutamine (Gibco, UK) inside a T-25 flask. Cells were detached with trypsin (ThermoFisher, UK) and cultivated in 24 well plates until reaching complete confluence. After this, a scratch was made into the middle of each well containing the cell monolayer using a 200 μL pipette tip to simulate a skin injury. The plate was washed 3 times with PBS to remove serum and dead cells before adding fresh DMEM without FBS. Bright field optical microscopy was used to acquire pictures of the scratches on each well at the same position over 72 h to evaluate migration from the borders of the scratch into the scratched region.

Samples were examined containing FMdEG-KTTKS at concentrations of 0.025 wt%, 0.0125 wt% and 0.00625 wt%. The negative control comprised the cell monolayer without lipopeptide whereas the positive control was the cell layer in the presence of conventional stimulants (TGF-β1, insulin and ascorbic acid).

## Results

### Self-assembly and conformation

With increasing concentration, peptide conjugates may undergo self-assembly into structures such as fibrils, nanotapes or micelles.^29–32^ Here we examine the self-assembly of FMdEG-KTTKS which is shown to form micelles in aqueous solution. We first tested for the existence of a critical micelle concentration (CMC) using the fluorescent probe 8-anilino-1-naphthalene sulfonic acid (ANS). The fluorescence of ANS is sensitive to the hydrophobicity of the surrounding environment, presenting little to no fluorescence in aqueous solution, but greatly enhanced and blue-shifted fluorescence in hydrophobic environments (here the interior of the micelle).^33,34^Fig. 1 shows the variation of the intensity of the peak maximum (*I*_0_) and wavelength (*λ*_0_) with concentration. The original spectra are presented in SI Fig. S3. There is a clear discontinuity in both quantities at the same concentration, around (0.04 ± 0.01) wt%, which is assigned as the possible CMC. We further investigated the conformation of FMdEG-KTTKS above the CMC using circular dichroism (CD) and FTIR spectroscopies and examined self-assembled nanostructures using dynamic light scattering (DLS), small-angle X-ray scattering (SAXS) and cryogenic transmission electron microscopy (cryo-TEM). The results are shown in Fig. 2. The CD spectrum shown in Fig. 2a is characteristic of an unordered peptide conformation.^35–37^ The FTIR spectra shown in Fig. 2b are also consistent with a disordered conformation due to the presence of a broad peak near 1650 cm^−1^.^38,39^ The peak at 1672 cm^−1^ is due to bound TFA counterions.^40–42^ The unordered conformation is consistent with the presence of micelles. Several structural methods were used to examine self-assembly above the CMC. Dynamic light scattering (DLS) was used to detect aggregates, Fig. 2c shows a representative intensity distribution, with a sharp peak corresponding to a hydrodynamic radius *R*_h_ = (4 ± 0.6) nm, which is consistent with the presence of micelles. Small-angle X-ray scattering (SAXS) provides further details on the structure of the aggregates. The data shown in Fig. 2d indicates well-defined form factor oscillations along with a flat low *q* intensity behavior for the least concentrated 0.5 wt% sample, characteristic features of a sphere form factor.^27^ The data were well described by a core–shell sphere form factor as detailed in the SI, which also provides a list of fit parameters (SI Table S1). The outer micelle radius is (2.4 ± 0.1) nm, which is consistent with DLS, considering the difference between hydrodynamic radii and thermodynamic radii from SAXS. Fig. 2e shows a typical cryo-TEM image with well-defined relatively monodisperse micelles with a size consistent with that obtained from DLS and SAXS. For the two higher concentration solutions studied (1 and 5 wt%, data shown in Fig. 2d) there is a broad structure factor peak in the SAXS data at low *q*, which could be described by including a hard sphere structure factor in the fits to the data (fit parameters in SI Table S1). For these higher concentrations however, the high *q* form factor oscillations are retained, indicating that the internal micelle structure is little changed (as also shown by the fit parameters, except *τ* which represents an overall scale factor of scattering contrast, dependent on concentration).

**Fig. 1:**
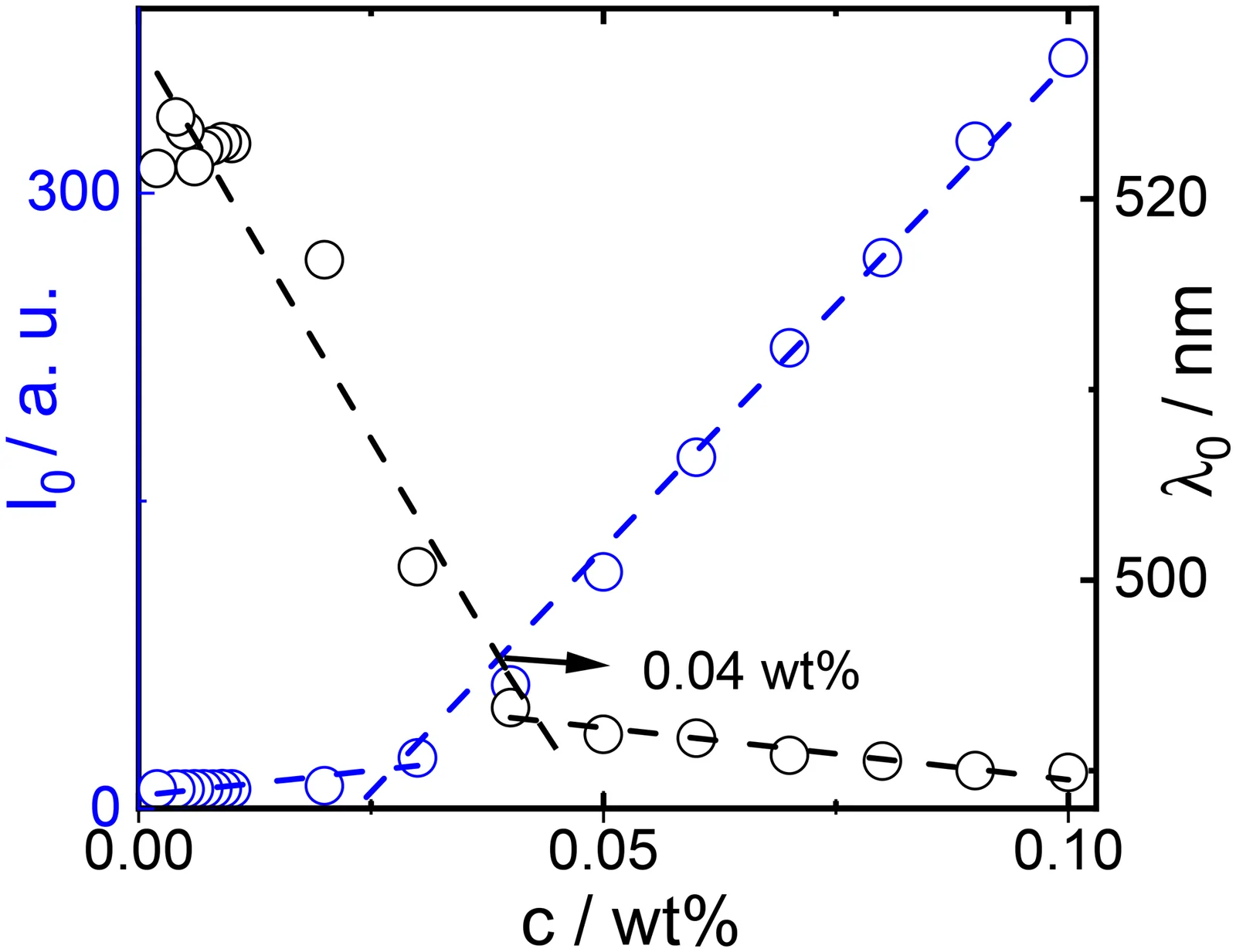
Maximum intensity, *I*_0_, and position, *λ*_0_, of the fluorescence emission peak of solutions containing ANS, plotted as a function of the FMdEG-KTTKS concentration.

**Fig. 2:**
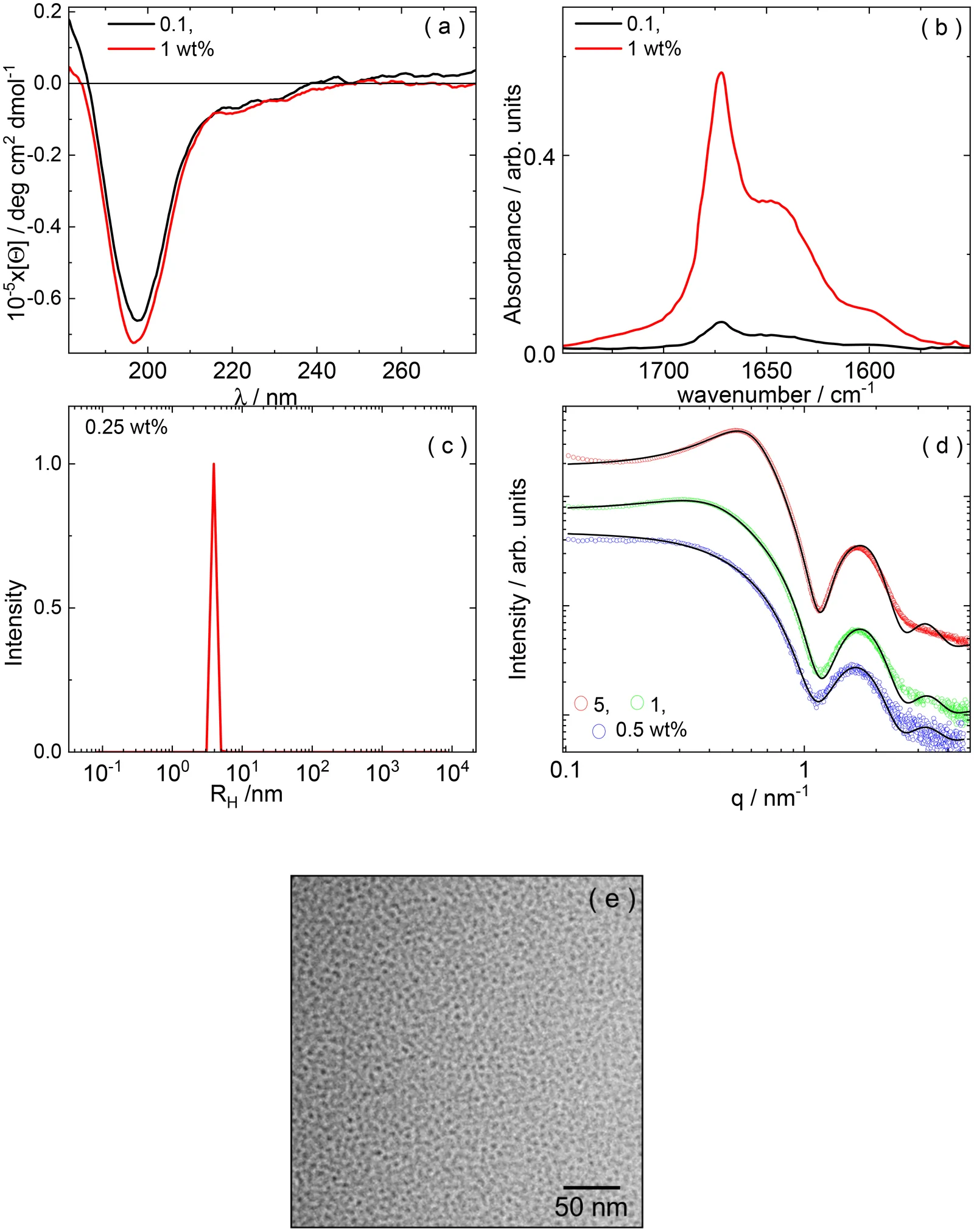
Conformation and self-assembly data. (a) CD, (b) FTIR, (c) DLS, (d) SAXS – measured data: symbols, fits: solid lines (curves offset for convenience) and (e) cryo-TEM image for aqueous solutions (at 20 °C) containing 0.1–5 wt% FMdEG-KTTKS (indicated in captions, concentration for cryo-TEM: 1 wt%). The parameters obtained from the fitting to the SAXS curves are listed in SI Table S1.

The micellar structure is notable in the context of cosmetic applications and can be contrasted with the β-sheet nanotape structure observed for C_16_-KTTKS^9^ or C_18_-KTTKS,^10^ which are stable across a wide pH range for C_16_-KTTKS, although micelles are observed at low pH.^12,13^ In contrast, SAXS measurements performed for FMdEG-KTTKS show features of micelle structures across a wide pH range, pH 2.6–pH 10 (SI Fig. S4). Only at higher pH 12–13 does the SAXS data show nanotape features, *i.e.* multiple orders of Bragg reflections from a lamellar repeat with a spacing *d* = 6.44 nm, which is consistent with a bilayer when the molecular length (estimated to be 4 nm) is considered.

### Cell culture and collagen detection assay

We accessed the cytotoxicity of FMdEG-KTTKS using cell viability assays with MTT. MTT assays measure the metabolic activity of a cell population due to the reduction of MTT by the mitochondria inside the cell membrane and can be interpreted as an overall indicator for cellular health.^43,44^Fig. 3 shows MTT data for a range of concentrations of FMdEG-KTTKS above and below the CMC, and for the conventional stimulants insulin, ascorbic acid and TGF-β1, which have been previously used to stimulate collagen production in fibroblasts.^5,45–47^ A statistically significant proliferative effect of the conventional stimulants is observed compared to controls incubated only with basal media, and there is a decrease in viability of around 30% for cells (compared to basal media) in the presence of FMdEG-KTTKS below 0.05 wt% (without statistical significance). Samples incubated with the lipopeptide at a concentration of 0.05 wt% or above presented a statistically significant decrease in the cell density, estimated by the MTT cell viability, when compared with the controls.

**Fig. 3:**
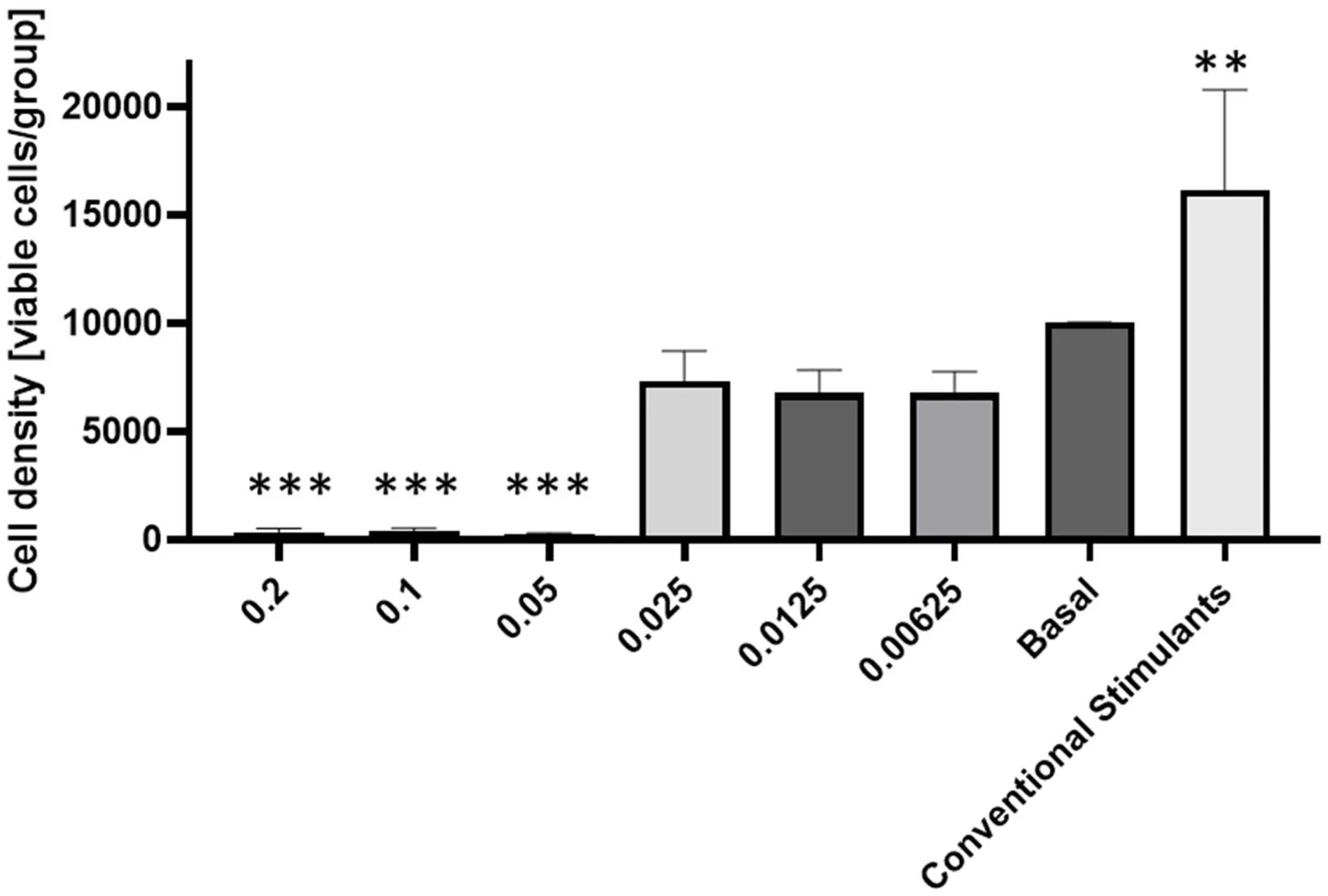
Average cell density of HDFa fibroblasts incubated for 72 hours with FMdEG-KTTKS from 0.2 to 0.00625%, conventional stimulants and controls containing only DMEM/F12 without any supplements (ANOVA, *n* = 3), **p < 0.01, ***p < 0.001.

Since compounds containing KTTKS such as Matrixyl™ are of interest due to collagen stimulation in cells, especially dermal fibroblasts, a collagen production assay using Picrosirius red was performed.^5,48,49^ Since the MTT assay indicated better tolerance for the lipopeptide at concentrations below the CMC, we evaluated the collagen production for three concentrations below the CMC. The results of this assay are shown in Fig. 4, while polarized optical microscopy (POM) images of the stained cells are shown in Fig. 5. An increase in collagen fibers is apparent from the accumulation of Picrosirius red and green/yellow birefringence shown in images from samples incubated with FMdEG-KTTKS for 72 hours (brightfield microscopy images are shown in SI Fig. S5). The quantitative data in Fig. 4a indicates a slight increase in collagen production by cells incubated with 0.025 and 0.0125% of FMdEG-KTTKS for all three concentrations studied, and a major increase for cells incubated with the conventional stimulants. Fig. 4b indicates the collagen production per cell, presenting a statistically significant increase for all concentrations of FMdEG-KTTKS evaluated.

**Fig. 4:**
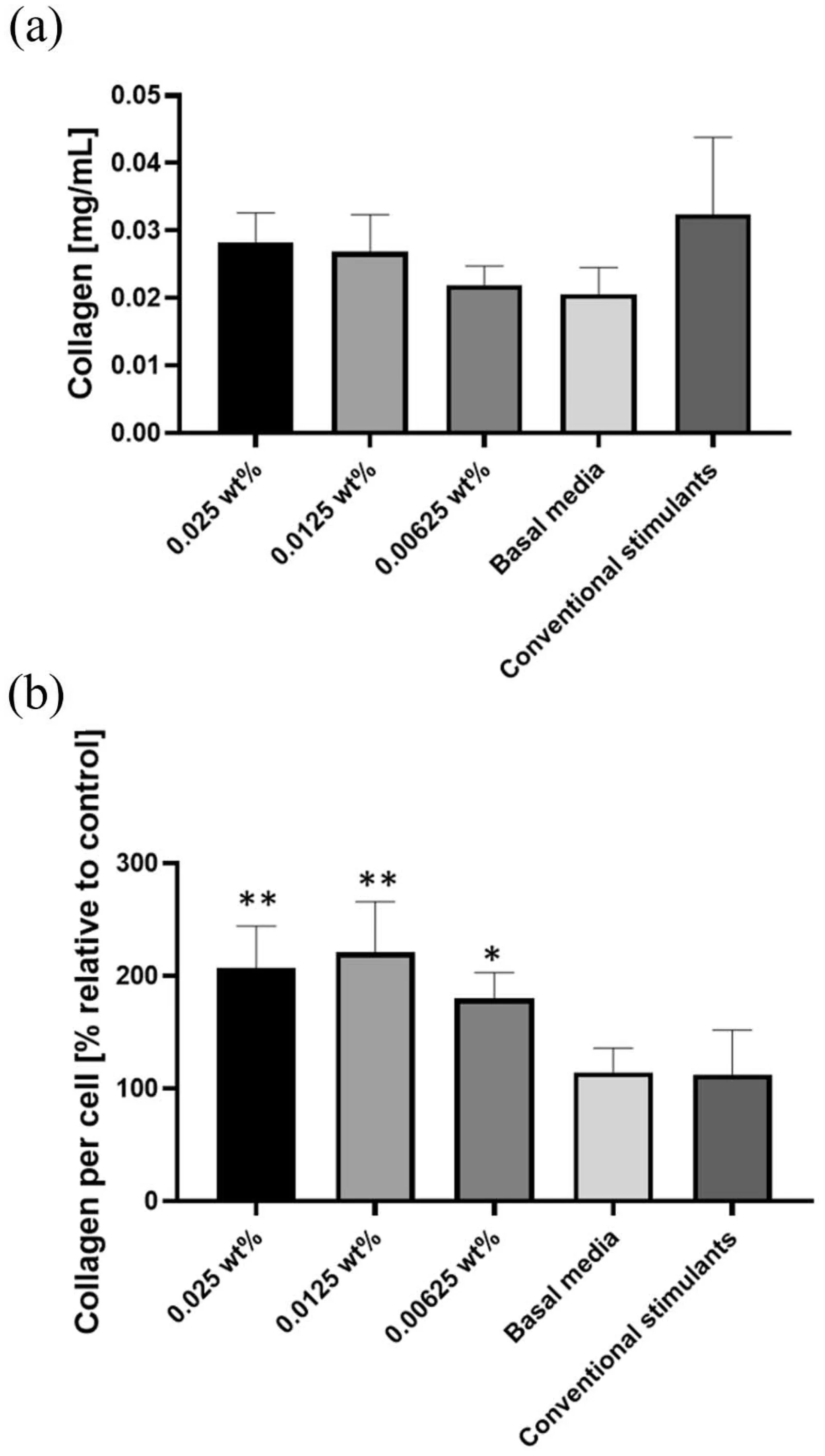
(a) Average amount of collagen [mg mL^−1^] produced by each well containing HDFa cells in the presence of FMdEG-KTTKS, conventional stimulants and basal media without any additives. (b) Average collagen production per cell [% relative to control] (ANOVA, *n* = 5), *p < 0.05, **p < 0.01.

**Fig. 5:**
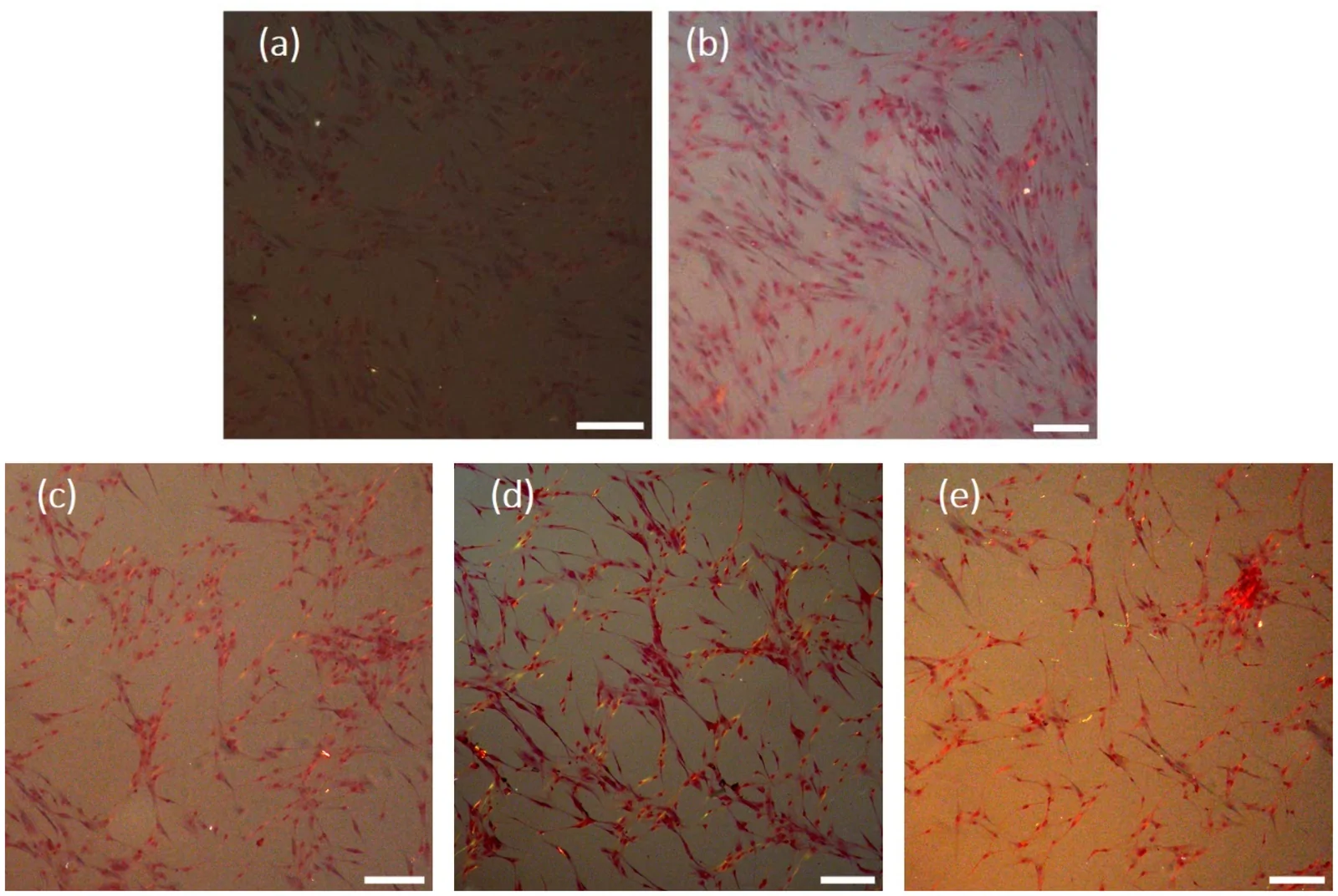
Polarized optical microscopy images of HDFa cells stained with Picrosirius red in the presence of (a) basal media, (b) conventional stimulants, (c) 0.00625 wt% FMdEG-KTTKS, (d) 0.0125 wt% FMdEG-KTTKS, (e) 0.025 wt% FMdEG-KTTKS. Scale bar = 200 μm.

An immunocytochemistry assay was performed on HDFa cells to compare the production of type I collagen and type III by confocal microscopy. The cells were also stained with DAPI and phalloidin-Texas red to reveal cell morphology. DAPI was used to stain the nucleus and to show that cells were viable at the time of fixation, and phalloidin was used to stain the cytoskeleton of cells, allowing the cytoplasm to be differentiated from the extracellular matrix and to better locate type I and type III collagen deposited on the plate. The micrographs shown in Fig. 6 show an increase in the production of collagen type I in samples incubated with 0.00625 wt% FMdEG-KTTKS. Specifically, increased production of collagen was observed in the extracellular matrix. A side-by-side comparison between HDFa cells incubated only in DMEM, conventional stimulant and FMdEG-KTTKS is shown in SI Fig. S6.

**Fig. 6:**
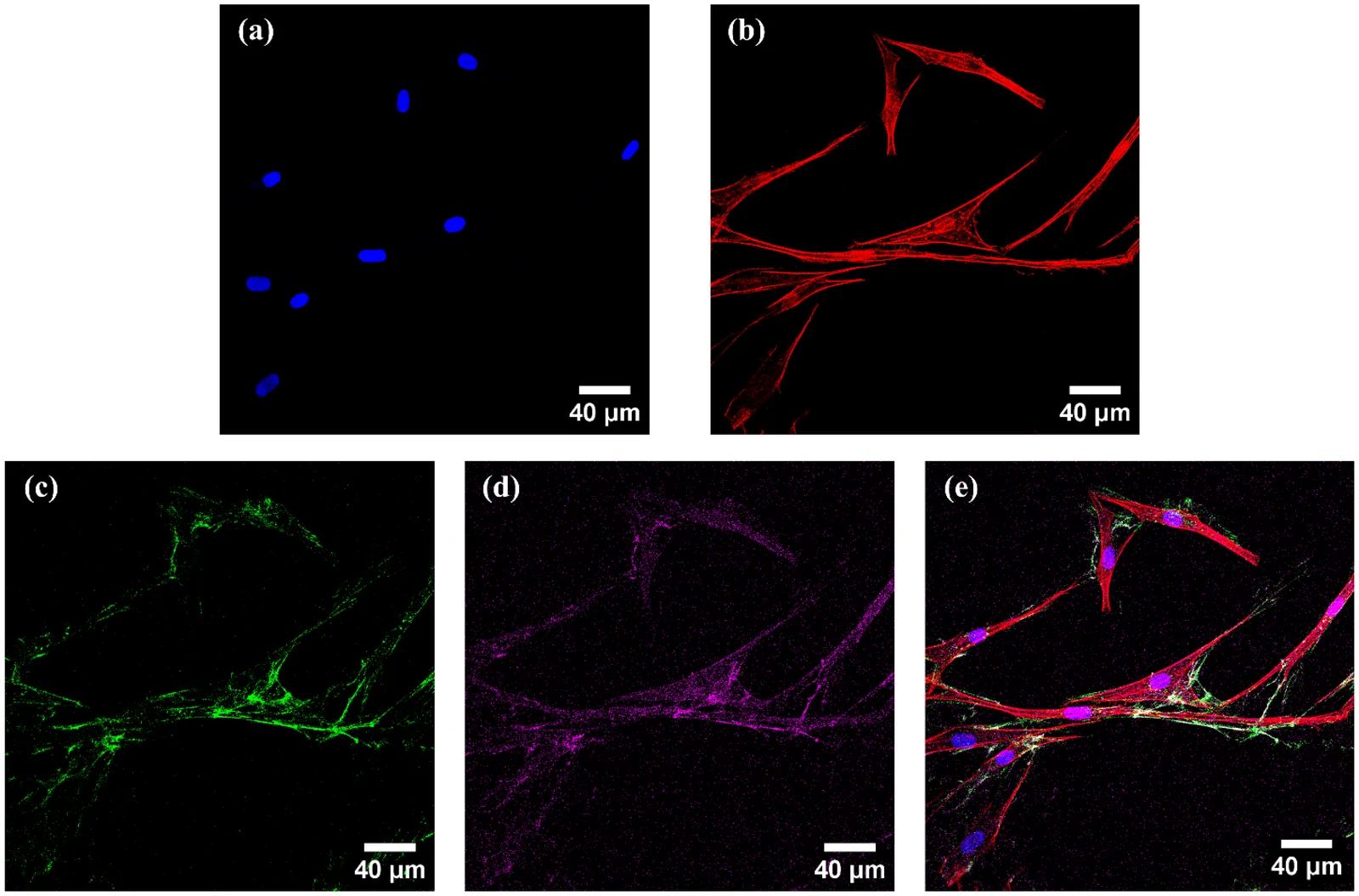
Immunocytochemistry imaged by confocal microscopy for HDFa cells incubated in FMdEG-KTTKS 0.0062 wt% for 72 hours, (a) nuclei stained with DAPI, (b) phalloidin red-stained actin in the cytoskeleton, (c) Alexa Fluor 488 labelling for type I collagen, (d) Alexa Fluor 647 labelling for type III collagen, (e) merged channels.

### Scratch assay

A scratch assay was performed to evaluate cellular migration over an “injury” made in a monolayer of fibroblasts, to simulate skin injury. This involved creating a cell monolayer on a multi-well plate; scratching a cell-free zone in the monolayer; and monitoring the recolonization of the cell free zone to quantity cell migration over a period of 72 hours. Microscope images following this process are shown in Fig. 7 for cells exposed to 0.00625 wt% FMdEG-KTTKS dissolved in media. During the healing process, migration of cells from the edges of the sectioned area to the middle of the decellularized area is observed after 24 h, visibly restoring the fibroblast layer after 72 h when compared to the control for which minimal, if any, migration of the fibroblasts present in the edges was observed even after 72 h (SI Fig. S7). Similar healing was observed for monolayers with 0.0125 wt% or 0.025 wt% FMdEG-KTTKS (data not shown). For the positive control (fibroblasts with conventional stimulants), repopulation of the scratched area after 72 h by fibroblasts was observed (SI Fig. S8).

**Fig. 7:**
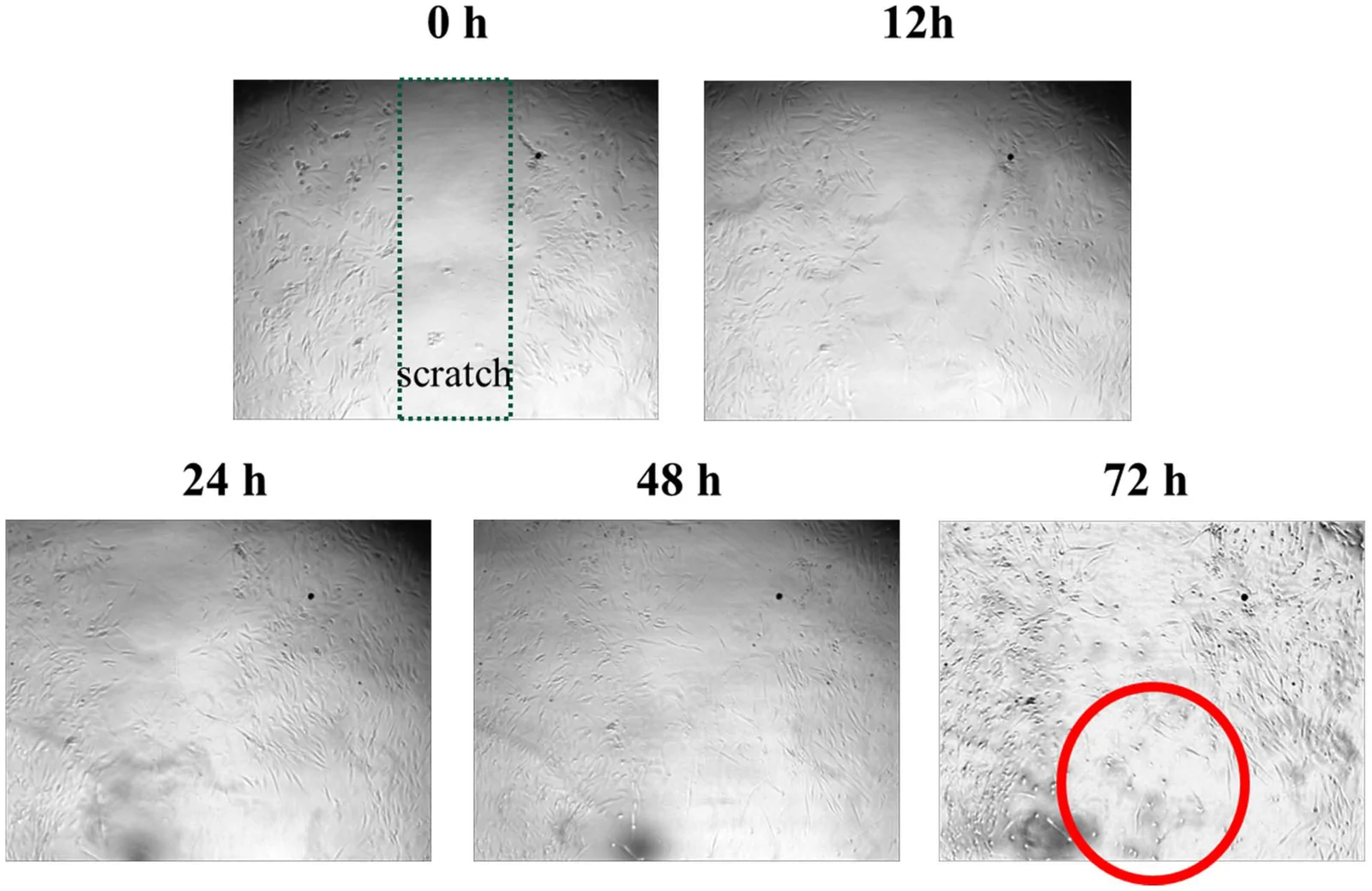
Scratch assay showing repopulation of scratched region on a plate surface by L929 cells in the presence of 0.00625 wt% FMdEG-KTTKS. Microscope images obtained at the time points indicated. The red circle in the image obtained after 72 h shows repopulation by fibroblasts with typical cell morphology.

### Anti-inflammatory activity

Nitric oxide (NO) is a signalling molecule involved in the pathogenesis of inflammation. Many cell types produce NO after stimulus during cellular injury or infections and NO can modulate inflammation in different tissues.^50^ The production of NO can be quantified using Griess reagent used to detect nitrite and nitrate in biological fluids, through the formation of red diazonium salts when nitrite reacts with a primary aromatic amine.^51^ During an inflammation process, NO is produced by cells and will quickly degrade into nitrite in the cell medium, which can be detected using the Griess reagent. We assessed the level of NO produced after challenge of macrophages with bacterial lipopolysaccharide (LPS) This test has previously been used to examine NO-scavenging activity of a palmitoyl tetrapeptide developed for wound healing applications.^52^ The data in Fig. 8 show that the nitrite levels for all three concentrations of FMdEG-KTTKS were significantly lower than for cells exposed to LPS and were comparable to the control comprising conventional stimulants used for cell migration and proliferation (1 mM ascorbic acid 0.025 ng mL^−1^ TGF-β1 and 0.01 mg mL^−1^ of insulin) in the absence of LPS, *i.e.* that the lipopeptide suppresses LPS-induced inflammatory NO production.

**Fig. 8:**
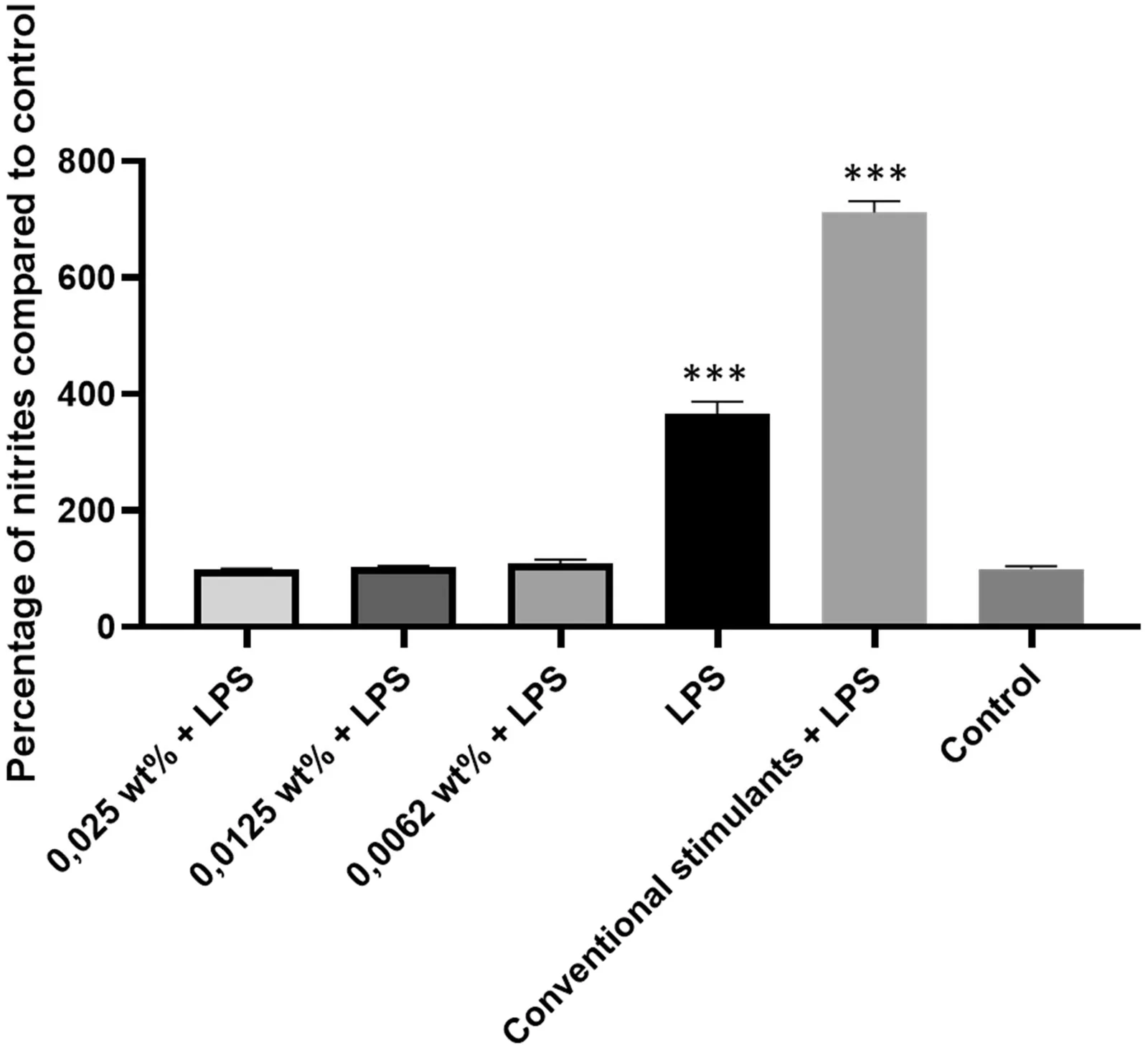
Griess assays performed using RAW 264.7 cells to detect of nitrites in the presence of FMdEG-KTTKS at the concentrations shown and in comparison to LPS alone and with stimulants or negative controls (with no lipopeptide, LPS or stimulants). ANOVA, *n* = 3, *p < 0.05, **p < 0.01, ***p < 0.001.

## Discussion

The designed bioactive collagen-stimulating peptide conjugate FMdEG-KTTKS undergoes self-assembly into spherical micelles above a CMC around 0.04 wt% as indicated by ANS fluorescence assays. The formation of micelles contrasts with previous reports on C_16_-KTTKS and C_18_-KTTKS which present extended nanostructures, *i.e.* long nanotapes.^9,10^ In the case of C_16_-KTTKS, micelles are only observed at very low pH^12,13^ or upon heating.^53^

As shown by SAXS, FMdEG-KTTKS forms micelles at room temperature over a range from pH 2–pH 10. This highlights the influence of the chain structure on the nanostructure formed. The di-ethylene glycol spacer constitutes a hydrophilic linker which enables FMdEG-KTTKS molecules to form micelles across a range of pH values where the lysine residues and C-terminus are expected to be charged.

In dilute solution, below 0.04 wt%, as indicated by the fluorescence assays in Fig. 1, FMdEG-KTTKS is mainly present in monomeric form. According to the Picrosirius red staining studies performed both in 96 well plate assays and imaged by polarized microscopy, under these conditions FMdEG-KTTKS may induce the production of collagen by dermal fibroblasts while preserving cell viability. Compared with C_16_-KTTKS based sequences, the cytocompatibility is improved since cell viability for C_16_-KTTKS is reduced by 40% or more, even at concentrations up to ten times lower (in the range 0.002–0.008 wt%) than used in the present work.^5^ The MTT assay shows that below the CMC, FMdEG-KTTKS does not present a statistically significant change in cell viability, indicating that there was also not a significant impact in the overall cell metabolism for HDFa cells incubated for 72 hours at low concentration. The Picrosirius red assays indicate that for concentrations of 0.025 wt% and below, HDFa cells exhibited a significant increase in collagen production per cell when compared to controls incubated either only in DMEM or together with common stimulants for collagen production. The substantial increase in cytotoxicity of FMdEG-KTTKS above the CMC, where the molecules are self-assembled into micelles, is notable and may be and indicative that the lipopeptide is better tolerated at the monomeric form when compared to aggregates. Whether this is just due to the increase in concentration of the ‘active compound’ and/or is influenced by the presentation of the peptide motif at high density on the surface of the micelles, remains an open question.

The Picrosirius red assays, both quantitative by absorbance assays and qualitative by polarized microscopy, indicates a significant increase in collagen when compared to controls, showing that FMdEG-KTTKS has a substantial collagen-stimulating effect, at low doses (lowest concentration 0.00625 wt% examined) which is beneficial for potential applications in skincare. The collagen production of FMdEG-KTTKS is decreased compared with the collagen production reported for C_16_-KTTKS, which resulted in a more than ten-fold increase in collagen/cell compared to basal media when incubated at 0.008 wt% lipopeptide.^5^ Although conventional stimulants presented a general increase in the total collagen produced per well, the overall collagen production is normalized by the number of cells present in the well, no statistically significant increase in the production of collagen per cell is detected. These results suggest that the overall increase in collagen production is mostly due to the proliferative increase of dermal fibroblasts under the influence of TGF-β1 and insulin.^54,55^

The yellow/red birefringence observed in polarized microscopy images incubated with the lipopeptide may be indicative of type I collagen production,^56^ which is the predominant collagen found in most tissues, including skin, tendons, bones and many other structures in the body.^57–59^ This is supported by immunocytochemical imaging using antibodies specific to collagen type I and type III, where a significant labelling of collagen type I around the cells was observed, with the presence of some type III collagen, although the relative ratio was not quantified. This effect contrasts with the more homogeneous collagen production in between the cells, mainly observed for samples incubated only in DMEM or conventional stimulants (SI Fig. S5), indicating an increase of collagen deposition in the extracellular matrix in the form of long and well defined fibers.

Apart from the cosmetic applications, skin injuries will often result in inflammatory processes due to tissue damage or infection from microorganisms present in the environment. Thus, we also evaluated how this new molecule could aid in the recovery of skin and prevent excessive inflammation, often a factor related to scarification and impaired recovery.^60,61^ The scratch assay is a simple model of “wound healing” using a fibroblast monolayer and can be used to assess cell repopulation in a 2D cell layer. Our data shows that FMdEG-KTTKS promotes cell repopulation in a decellularized region to a greater extent than controls incubated only in media without any conventional stimulants. This is an improvement in comparison to a monolayer of cells incubated only in DMEM/F12 media, which still presented a large decellularized area even 72 hours after scarring, due to the lack of any growth factors or molecules that would stimulate migration and proliferation. The positive control for the wound healing scratch assay applied the same conventional stimulants previously used for the collagen assays, with a complete recovery of the simulated wound due to the proliferative characteristics of TGF-β1.

Skin injuries are prone to generate inflammation due to many different factors, and FMdEG-KTTKS also shows remarkable anti-inflammatory activity. The Griess reagent assays show significantly less nitrite production in macrophages challenged with bacterial LPS in the presence of the lipopeptide compared to controls, with NO levels comparable to those for HDFa cells not exposed to LPS. The anti-inflammatory effect may be attributed to the influence of the farnesyl group in FMdEG-KTTKS, for which anti-inflammatory effects have been noted previously, including regulation of the response to oxidative stress and controlling cell migration.^62–64^ Our findings suggest that FMdEG-KTTKS may not only aid with collagen production and cell repopulation after injury, but also act as a potential anti-inflammatory molecule.

## Conclusions

We present a comprehensive biophysical and biological characterization of a novel matrikine lipopeptide, FMdEG-KTTKS. At higher concentrations above the CMC, this lipopeptide self-assembles into small micelles a few nanometers in diameter, as indicated by SAXS, cryo-TEM and DLS. FTIR and circular dichroism assays show that the peptide adopts an unordered conformation, which may be due to the presence of the attached lipid and spacer. The branched lipid chains in FMdEG-KTTKS are likely to lead to a larger area per chain than for unbranched lipids, and hence a lower surfactant packing parameter, favouring micelles in contrast to the lamellar structure observed (at low temperature) for C_16_-KTTKS, with an unbranched saturated lipid chain.

Microscopy images using fluorescence indicated that most collagen produced was present in the ECM, consisting of type I collagen, with some type III also, another important component of the interstitial matrix that supports the skin tissue and provides mechanical support in helping to maintain skin homeostasis during recovery from injury.^65^ A high ratio of collagen I/III is a desirable trait for anti-aging treatments,^66^ and should be quantified in future work to further support the promise of FMdEG-KTTKS for skincare applications.

Overall, the biological assays indicate that FMdEG-KTTKS has collagen-inducing properties allied to anti-inflammatory effects and, at sufficiently low concentration, low cytotoxicity. This is a desirable combination for the application of this compound as a skin cosmetic or even for possible clinical applications, for which excessive proliferation or cytotoxicity may be undesirable.

The isoprenoid chain and linker connection in FMdEG-KTTKS may promote micelle formation. The micellar structure is interesting for cosmetic applications as it is typically correlated to surface activity (surfactancy).^67,68^ In addition delivery in micelle form may enable permeation of the bioactive lipopeptide into tissues including the stratum corneum. This is currently under investigation and will be presented in a future report.

## Conflicts of interest

The authors declare no competing financial interests. The research reported herein is the basis for a patent application.^69^

## Supplementary Material

NR-OLF-D6NR03203F-s001

## Data Availability

The data is available upon reasonable request from the authors. Supplementary information (SI): HPLC and ESI-MS molecular characterization, ANS fluorescence spectra, SAXS data as a function of pH, bright field microscopy images of Sirius red stained cells, immunocytochemistry images including controls, microscopy images for controls for scratch assays, table of SAXS fit parameters. See DOI: https://doi.org/10.1039/d6nr03203f.
