# Supplementary material for: Collagen-stimulating activity of a pentapeptide conjugate and self-assembly into micelles
Source: Nanoscale. 2026 Oct 26. Online ahead of print. doi: 10.1039/d6nr03203f (PMC13726402; doi:10.1039/d6nr03203f)
Supplement: Supplementary file 1 [file NR-OLF-D6NR03203F-s001.pdf]

Supplementary Information (SI) for Nanoscale.
This journal is © The Royal Society of Chemistry

Supporting Information

Collagen Stimulating Activity of a Pentapeptide Conjugate and Self-Assembly into Micelles

Lucas de Mello,[1] Valeria Castelletto,[1] Jani Seitsonen,[2] Ian W Hamley[1]

[1] *School of Chemistry, Food Biosciences and Pharmacy, University of Reading, Whiteknights, Reading RG6 6AD, U.K.*

[2] *Nanomicroscopy Center , Aalto University, Puumiehenkuja 2, FIN-02150 Espoo, Finland*

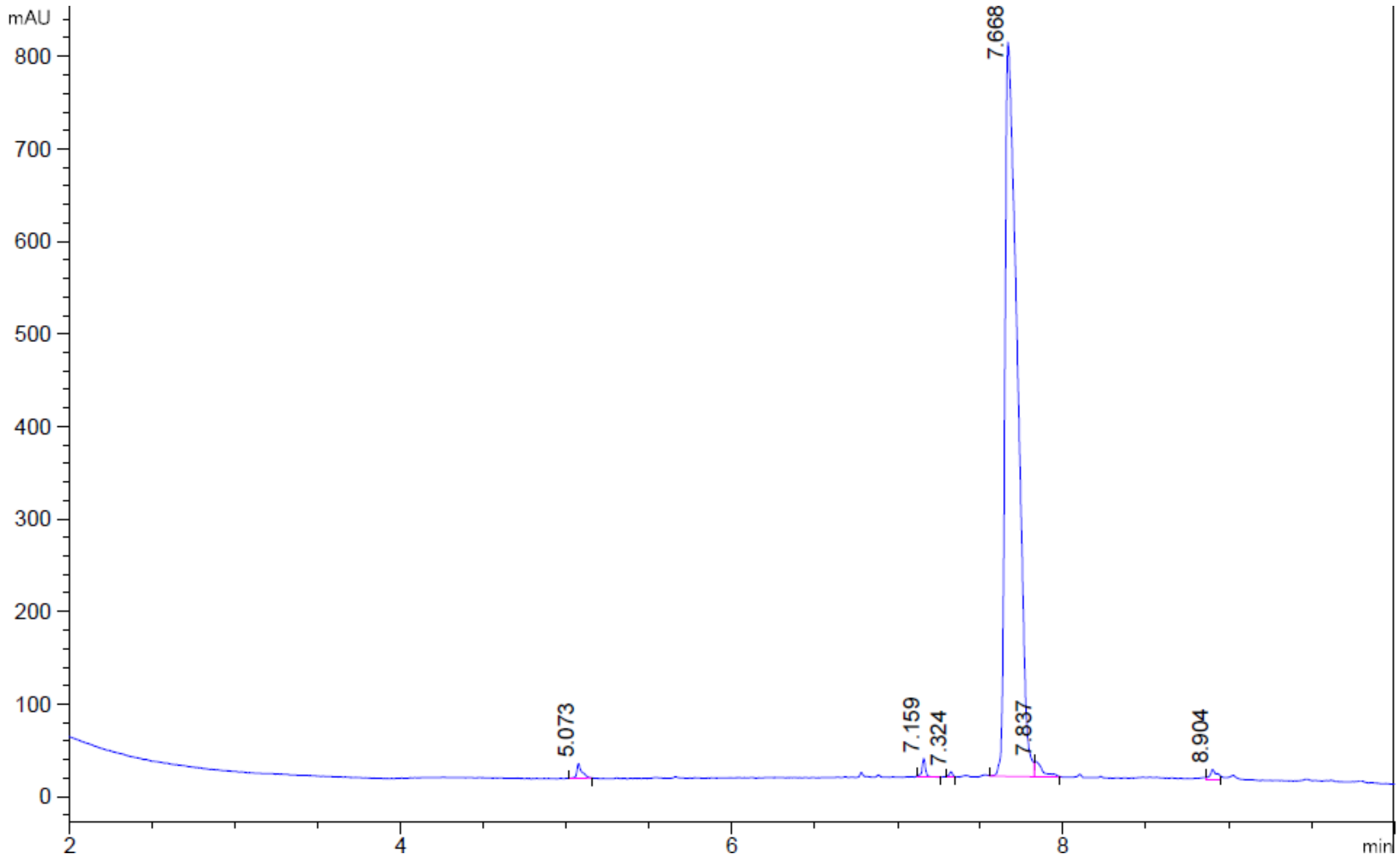

#	Meas. Ret.	Area	Area %
1	5.073	33.269	0.776
2	7.159	29.830	0.696
3	7.324	8.629	0.201
4	7.668	4124.450	96.263
5	7.837	58.476	1.365
6	8.904	29.930	0.699

Figure S1. HPLC data for one batch of FMdEG-KTTKS, purity is 96.3% (indicated in table).

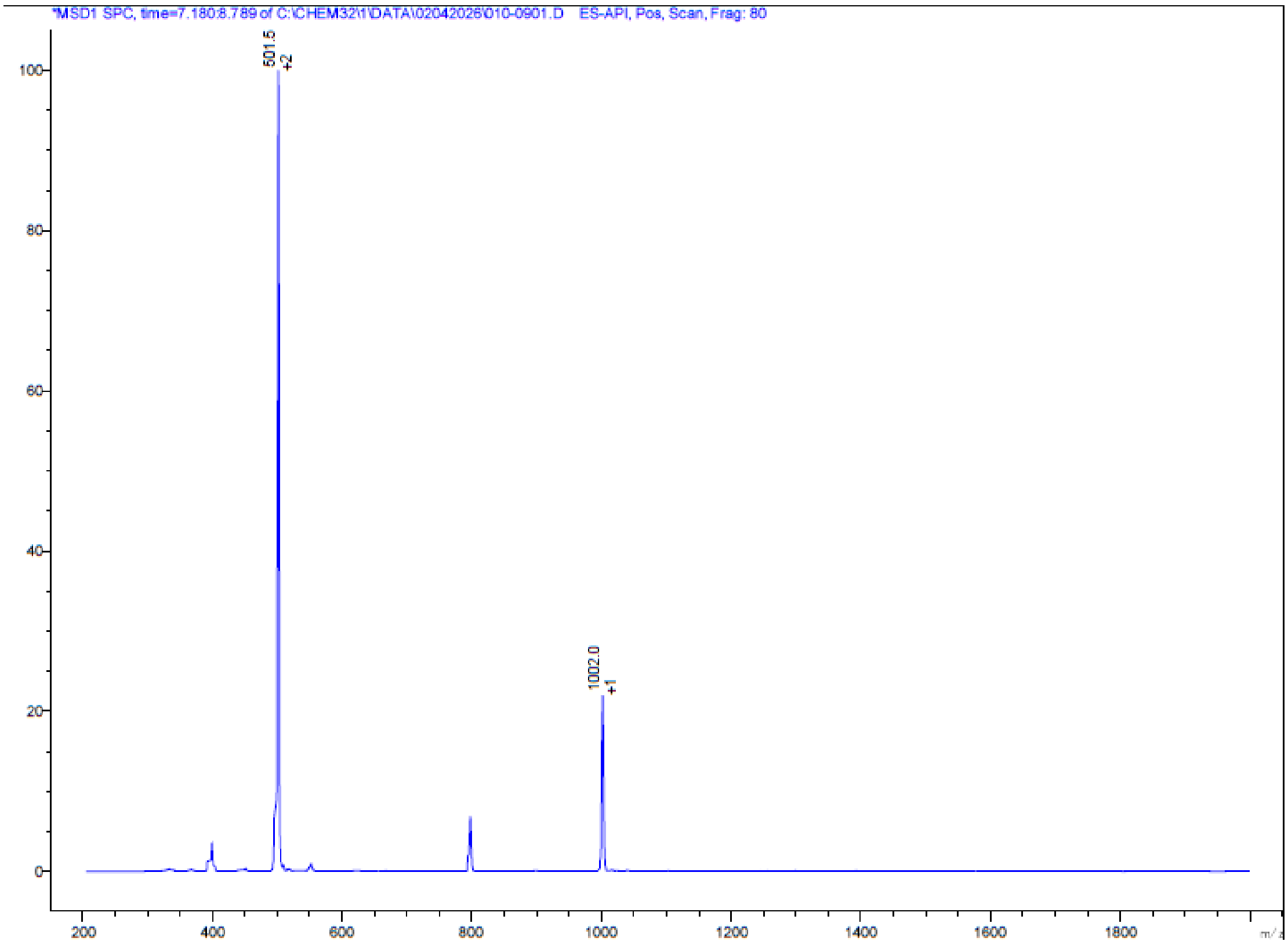

Figure S2. ESI-MS data for same batch of FMdEG-KTTKS as data in Figure S1.

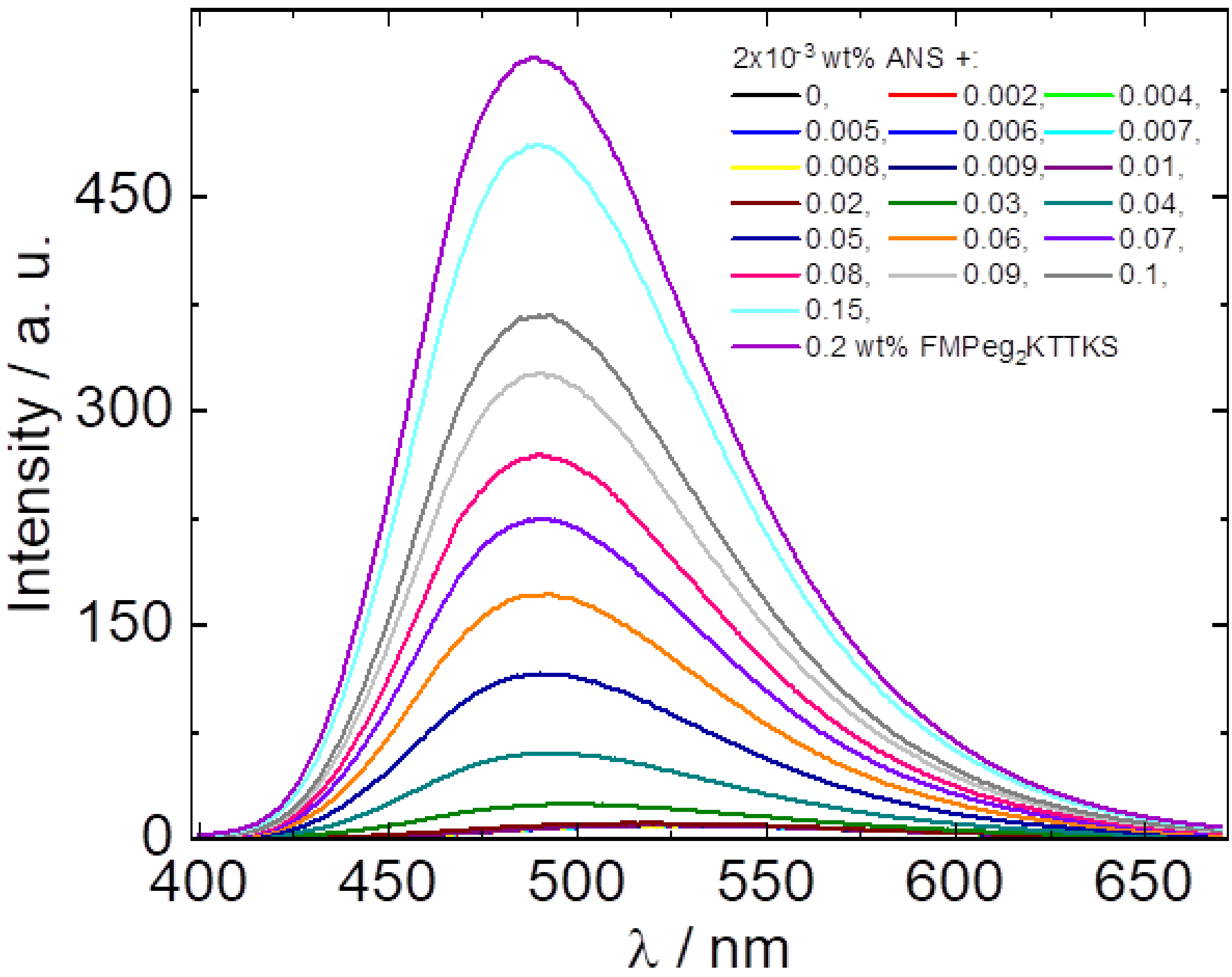

Figure S3. ANS fluorescence intensity curves used to construct the plot in Figure 1.

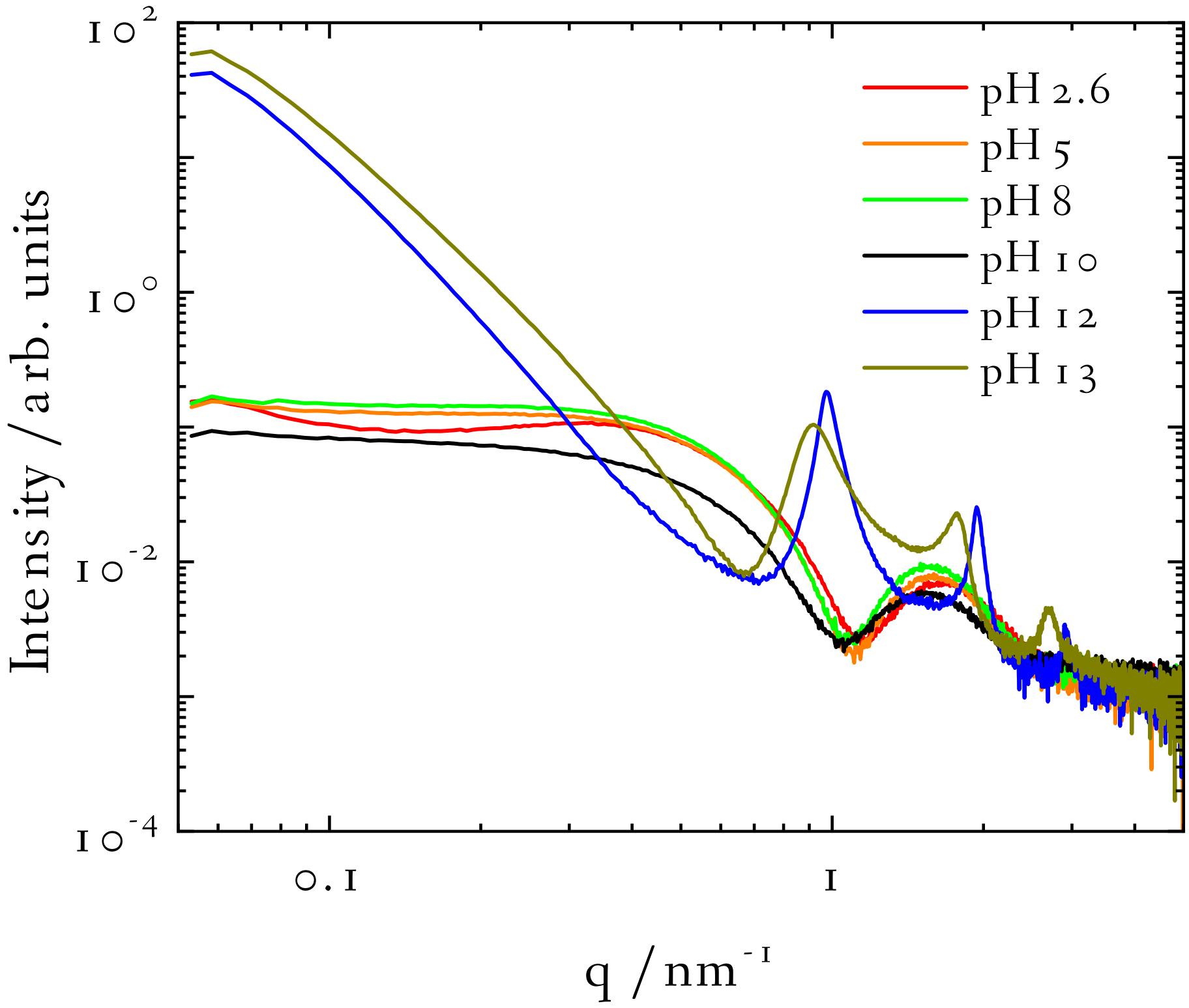

Figure S4. SAXS data for 1 wt% solutions of FMdEG-KTTKS at the pH values indicated.

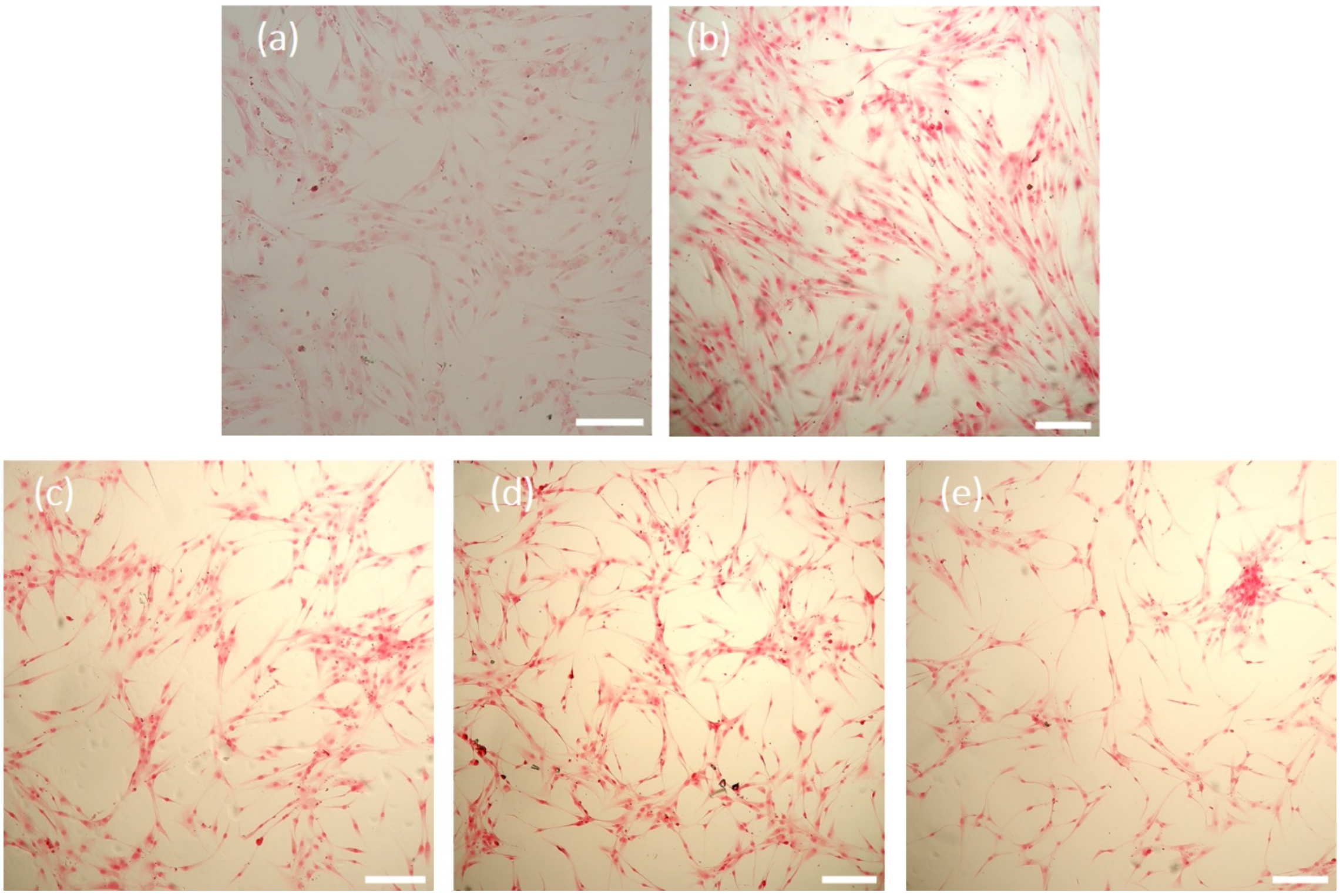

Figure S5. Brightfield optical microscopy images of HDFa cells stained with Picrosirius red in the presence of (a) Basal media, (b) Conventional stimulants, (c) 0.00625 wt% FMdEG-KTTKS, (d) 0.0125 wt% FMdEG-KTTKS, (e) 0.025 wt% FMdEG-KTTKS. Scale bar = 200 μm.

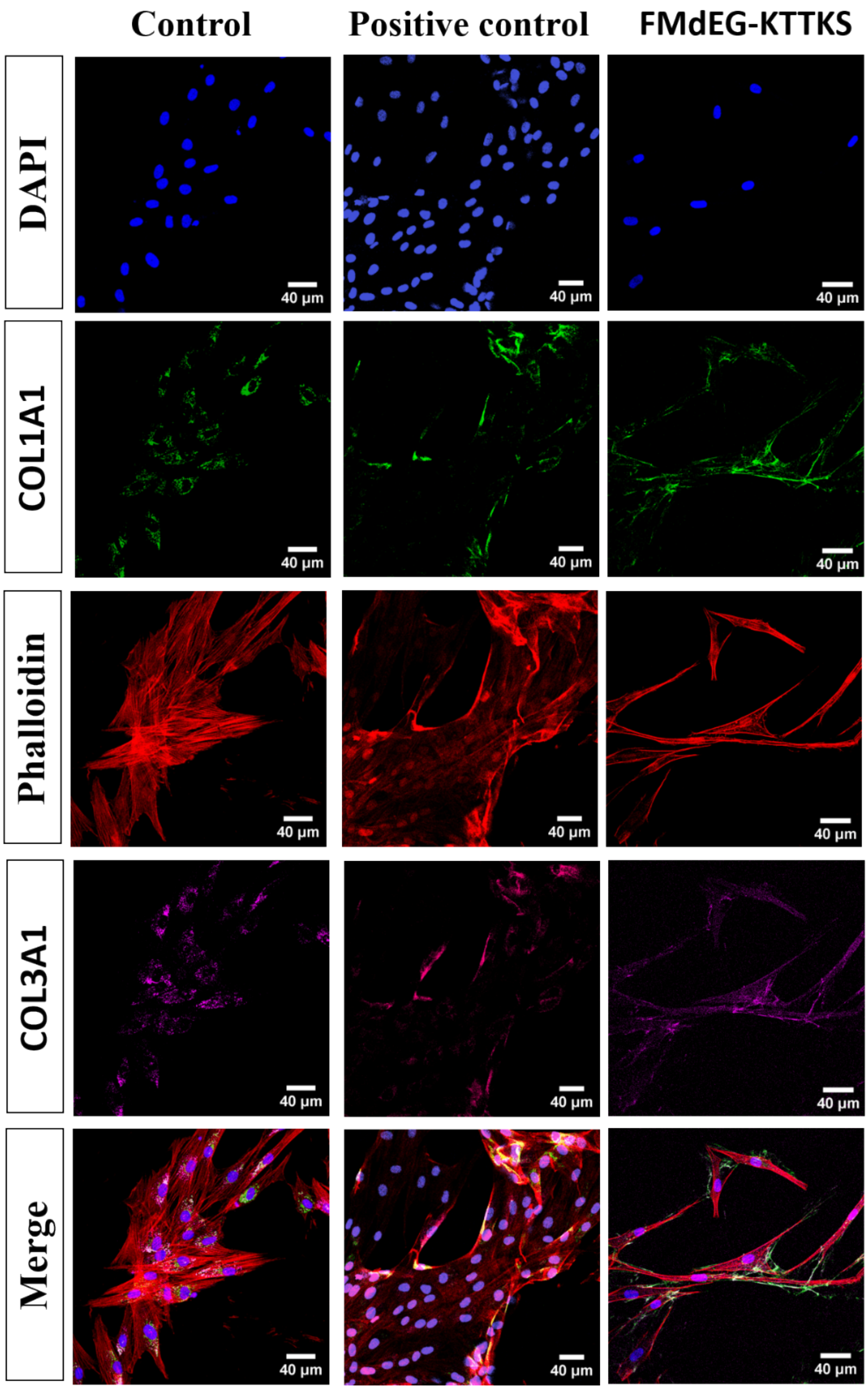

Figure S6. Immunocytochemistry images for **Control**: HDFa cells incubated only in basal DMEM media, **Positive Control**: conventional stimulants containing TGF-b1, insulin and citric acid, **FMdEG-KTTKS**: 0.00625 wt%. solution Each row represents different labelling, with the nucleus stained with DAPI (blue), Phalloidin red staining actin in the cytoskeleton (red), Alexa Fluor 488 labelling for type I collagen (green) and Alexa Fluor 647 labelling for type III collagen (magenta). The last row shows a merge of all four channels.

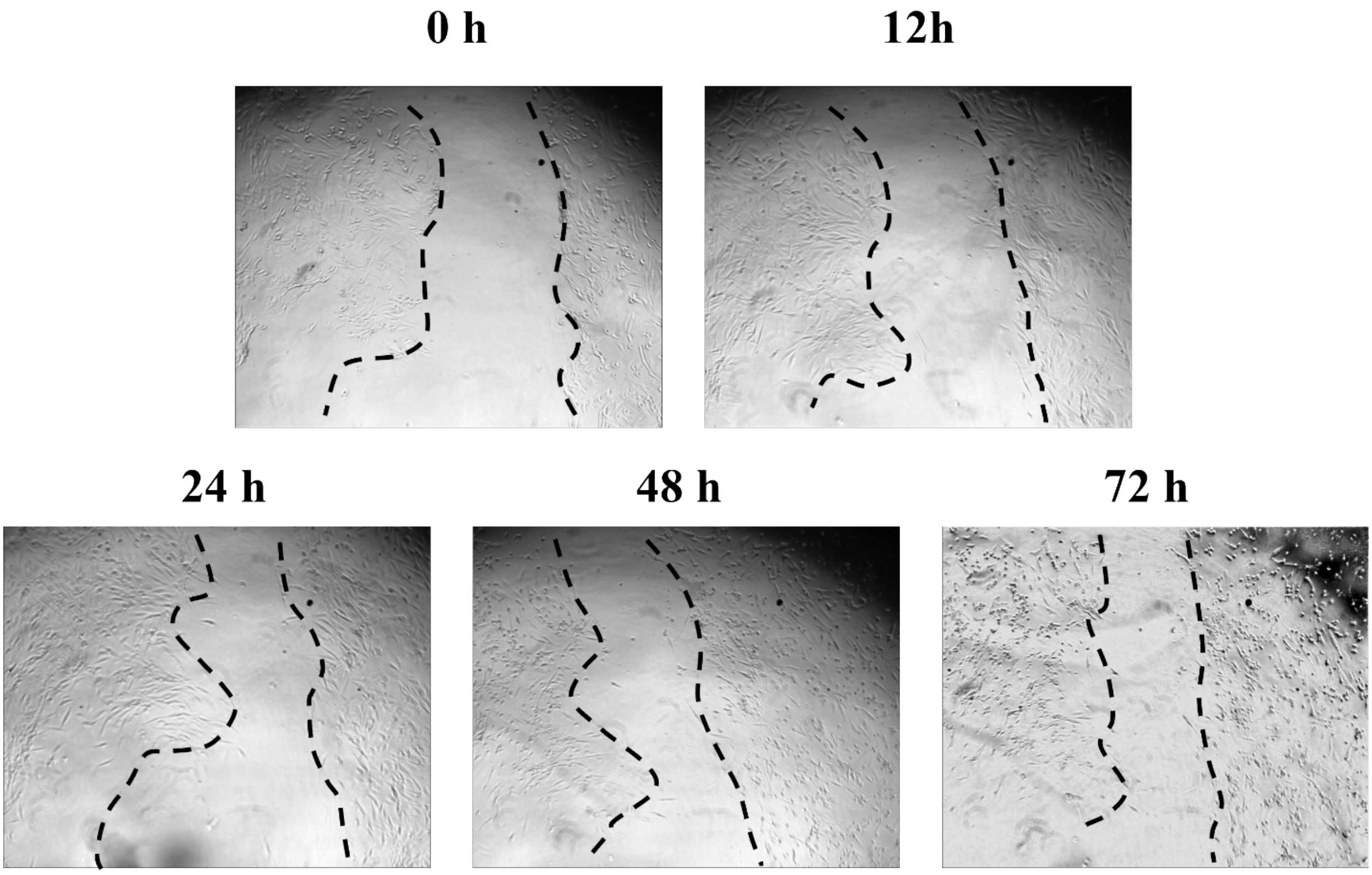

Figure S7. Scratch assay showing repopulation of scratched region shown on a plate surface by human dermal fibroblast cells as controls without FMdEG-KTTKS or collagen stimulants at the time points indicated. For better visualization, dashed lines are placed at the borders of the 'wound' in the cell monolayer.

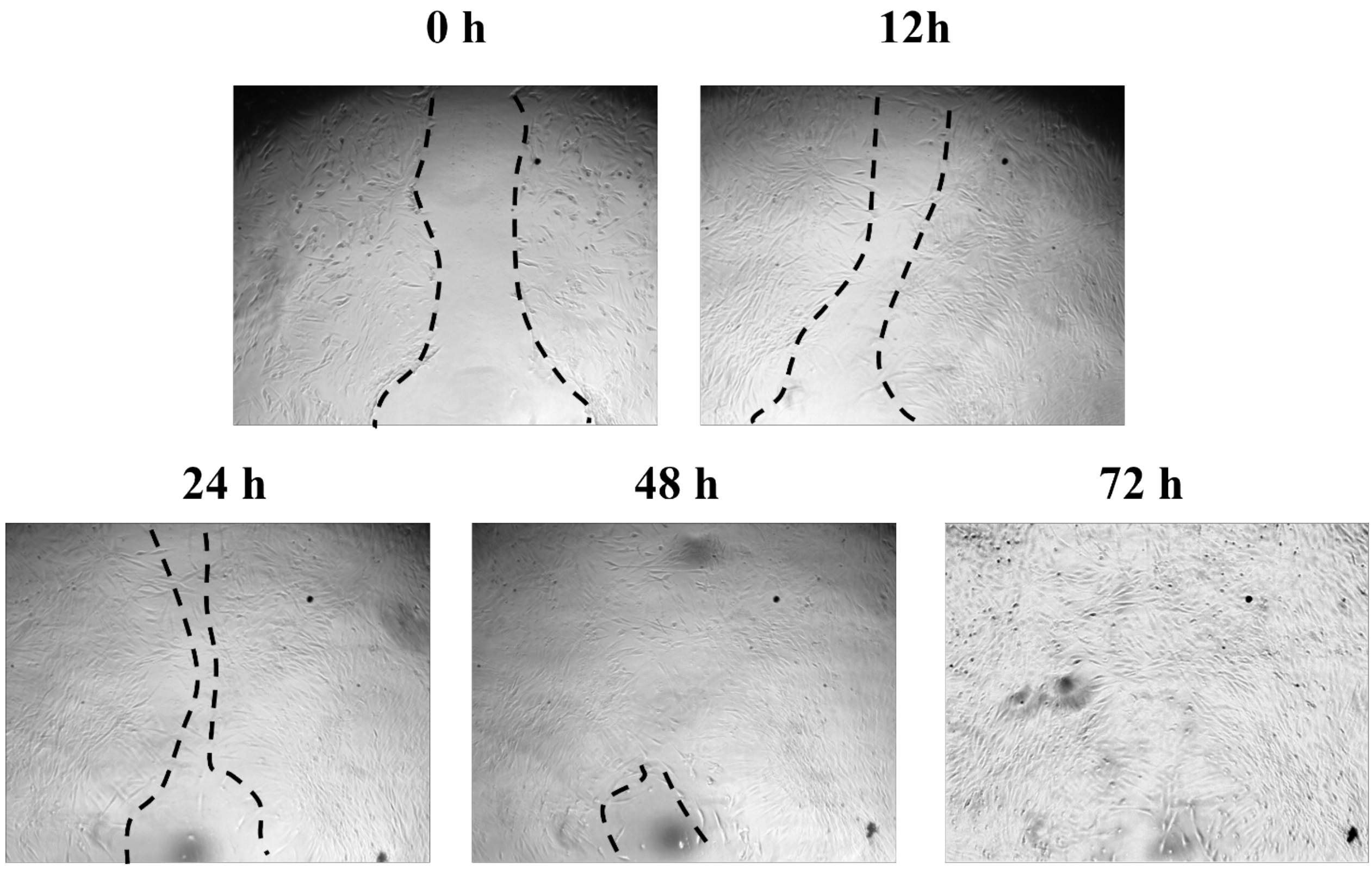

Figure S8. Positive control scratch assays showing the repopulation of a scratched region by human dermal fibroblast cells incubated with conventional stimulants (TGF-b1, insulin and ascorbic acid) without FMdEG-KTTKS at the time points indicated. For better visualization, dashed lines are placed at the borders of the simulated wound in the cell monolayer.

Table S1. Parameters extracted from the fitting of the SAXS data in Figure 2d, using the form the form factor for a spherical shell, together with the structure factor for a hard sphere.

	0.5 wt% FMDEG-KTTKS	1 wt% FMDEG-KTTKS	5 wt% FMDEG-KTTKS
R_1 / nm	2.34 ± 0.64	2.40 ± 0.57	2.60 ± 0.52
R_2 / nm	1.8	1.8	1.8
μ / cm^{-1}	-4.8×10^{-2}	-3.8×10^{-2}	-3.8×10^{-2}
□ / cm^{-1}	3.7×10^{-3}	6.1×10^{-3}	1.3×10^{-2}
R_{HS} / nm	--	6.6	4.8
f	--	6×10^{-2}	1.9×10^{-2}
C	5.7×10^{-4}	1×10^{-3}	4×10^{-3}

Key. Spherical Shell Form Factor: overall radius of the spherical shell, R_1, radius of the core, R_2, scattering contrast of the inner core, μ, scattering contrast of the shell, □. **Hard Sphere Structure Factor:** hard sphere radius, R_{HS}, volume fraction of hard spheres, f. Background, C.
